# Wide crossing diversify mitogenomes of rice

**DOI:** 10.1186/s12870-020-02380-w

**Published:** 2020-04-15

**Authors:** Weilong Yang, Jianing Zou, Jiajia Wang, Nengwu Li, Xiaoyun Luo, Xiaofen Jiang, Shaoqing Li

**Affiliations:** State Key Laboratory of Hybrid Rice, Key Laboratory for Research and Utilization of Heterosis in Indica Rice of Ministry of Agriculture, Engineering Research Center for Plant Biotechnology and Germplasm Utilization of Ministry of Education, College of Life Science, Wuhan University, Wuhan, 430072 China

**Keywords:** Mitochondria, Rice, Genomic rearrangement, Maternal inheritance, Nucleo-cytoplasmic interaction

## Abstract

**Background:**

In most angiosperms, the inheritance of the mitochondria takes place in a typical maternal manner. However, very less information is available about if the existence of structural variations or not in mitochondrial genomes (mitogenomes) between maternal parents and their progenies.

**Results:**

In order to find the answer, a stable rice backcross inbred line (BIL) population was derived from the crosses of *Oryza glaberrima*/*Oryza sativa//Oryza sativa.* The current study presents a comparative analysis of the mitogenomes between maternal parents and five BILs. There were recorded universal structural variations such as reversal, translocation, fusion, and fission among the BILs. The repeat-mediated recombination and non-homologous end-joining contributed virtually equal to the rearrangement of mitogenomes. Similarly, the relative order, copy-number, expression level, and RNA-editing rate of mitochondrial genes were also extensively varied among BILs.

**Conclusions:**

These novel findings unraveled an unusual mystery of the maternal inheritance and possible cause for heterogeneity of mitogenomes in rice population. The current piece of work will greatly develop our understanding of the plant nucleo-cytoplasmic interaction and their potential role in plant growth and developmental processes.

## Background

A coordinated nucleo-cytoplasmic interaction plays a crucial role in the plant development [1, 2]. However, inter- or intraspecies hybridization in plants may lead to the reshuffling of the nuclear genome and eventually might disturb the equilibrium of cytonuclear interactions. Therefore, maintenance of a coordinated nucleo-cytoplasmic interaction would determine the growth, development, and even survival of the plants [3, 4]. Unlike the nuclear genome, mitochondria and chloroplast generally follow a uniparental inheritance in plants [5, 6]. It is considered that except some special cases, maternal inheritance appears to be the predominant mode of mitogenome transmission in plants especially angiosperms, and cycads and gnetophytes (gymnosperms) [7–9]. Therefore, it becomes very essential to understand the mechanism of acclimatization of the maternally inherited mitogenomes with the reshuffled nuclear genome to retain a determined nucleo-cytoplasmic interaction during plant evolution and crop domestication. Here, we speculate that the mitogenome may have evolved a special genetic mechanism in order to cope with the frequent rearrangement of nuclear genomes in nature. Interestingly, previous reports have also documented the apparent differentiation of mitogenome in plant populations including *Silene vulgaris*, *Cucurbitaceae,* and *Actinidia* families [10–13]. It is proposed that mitogenome possibly transmits in a dynamic way in the filial progenies to reinstate a balance of mitonuclear interaction in plants. Otherwise, it may be hard to explain the rich diversity of mitogenomes within a plant species population only by means of traditional maternal inheritance.

Rice, as a model cereal crop, shows a typical example of maternal inheritance. It is generally considered that indica rice was primarily originated from wild rice *Oryza rufipogon* in the middle of the Yangtz river, which was later domesticated into japonica rice in Northern China [14, 15]. However, recent genomic research suggests that both indica, as well as japonica rice, were originated from *O. rufipogon* simultaneously [16, 17]. It indicates that the mitogenomes of indica and japonica varieties are well-differentiated and show a narrow diversity in each subspecies if their mitogenomes strictly persist in stable maternal inheritance. However, rice population cannot only be differentiated into a group of subpopulations at nuclear level [18] but also significantly at mitogenome level. The mitogenomes of 224 worldwide landraces of rice were analyzed using 32 mitotype-specific molecular markers [19], all the landraces were classified into 20 subgroups, and the indica and japonica lines were found evenly distributed in each clade (Supplementary Fig. S1), implying a strong differentiation of rice mitogenomes [15, 17]. Such characteristics make rice as an apt model plant for the investigation of mitogenome inheritance and evolution patterns.

In order to prove this hypothesis, we developed a stable rice BC_2_F_12_ backcross inbred line (BIL) population which inherited their mitochondrial genomes from the same maternal parent (*O. glaberrima* × *O. sativa* 93–11). Subsequently, high-quality draft assemblies of mitogenomes were constructed and assayed for the genomic structure, gene content, DNA rearrangement, repeat sequences, as well as gene expression and RNA-editing among the maternal line and BILs with diverse genotypes. It was found that structure and organization of mitogenome, gene copy-number, expression, and RNA editing all varied to certain degrees among the BILs. The current novel findings revamp the traditional concept of maternal inheritance and greatly extend our understanding of the plant nucleo-cytoplasmic interactions.

## Results

### Backcross inbred lines derived from crosses of *O. glaberrima* and *O. sativa* showed different mitotypes from the maternal line

**Fig. 1:**
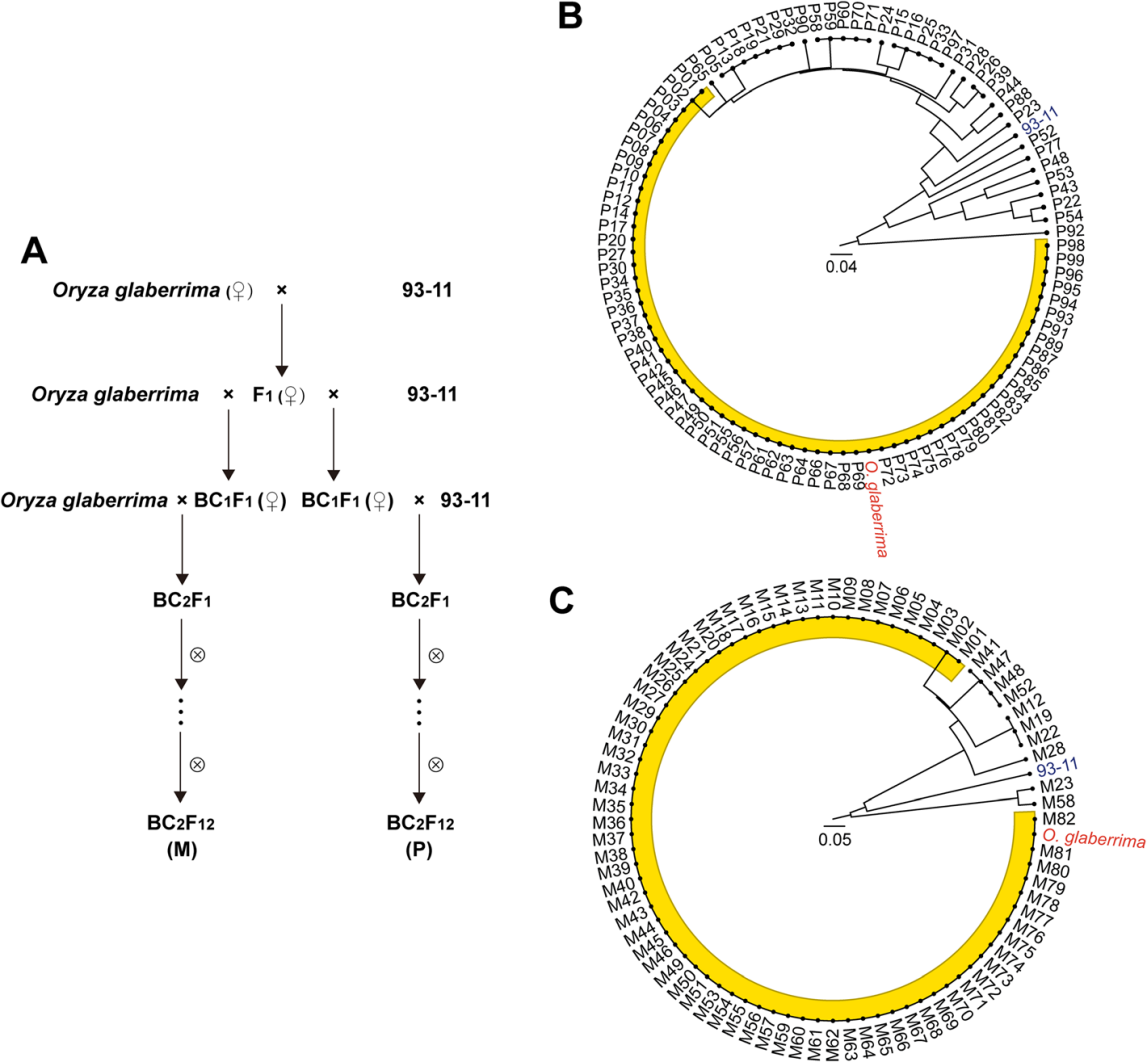
Construction and detection of structural variation of mitogenomes in backcross inbred lines. **a**. Schematic diagram for the construction of backcross inbred lines. **b, c**. Clustering analysis of *O. glaberrima*/93–11//93–11 and *O. glaberrima*/93–11//*O. glaberrima* BC_2_F_12_ BIL lines respectively, based on the mitochondria-specific molecular markers

In the quest for the existence of dynamic inheritance of rice mitogenome, two sets of BC_2_F_12_ backcross inbred lines (BIL) were constructed by crossing *O. sativa* 93–11 with *O. glaberrima* line 675 as maternal line, and then introgressed using 93–11 and *O. glaberrima* as the paternal line, respectively, in an attempt to simulate the natural evolutionary process of rice (Fig. 1a). Finally, 99 BILs from *O. glaberrima*/93–11//93–11 (P) and 82 BILs from *O. glaberrima*/93–11//*O. glaberrima* (M) were developed. These BILs were further analyzed for DNA polymorphism in the mitogenomes using mitochondria-specific molecular markers [19]. Although most of the paternal, as well as maternal BILs, were found to be grouped together with *O. glaberrima*, about 35.4% of the paternal (Fig. 1b) and 12.2% of the maternal BILs (Fig. 1c) were apparently distinct from their maternal parent *O. glaberrima*. It indicates that the mitogenomes are differentiated among different BILs, despite their inheritance from the same maternal line.

### Assembly of the mitogenomes of *O. glaberrima* and BILs

In order to decipher the structural variation of the mitogenomes, the BILs from the same cross (*O. glaberrima*/93–11//93–11) were selected for subsequent analysis owing to their distinct plant stature (Supplementary Fig. S2), differentiated mitotypes (Fig. 1b), and genotypes (Supplementary Fig. S3) verified with over 20-fold genomic sequencing (Supplementary Table S1).

In order to ascertain the accuracy of assembly, initially, a draft assembly of mitogenome of *O. glaberrima* was constructed using PacBio along with Illumina sequencing (Supplementary Table S2, S3). It contained two scaffolds, with minimum estimates of genome size of 402,174 bp, and 44.0% GC content (Table 1). The mitogenome of *O. glaberrima* was compared with that of Nipponbare (a high-quality reference mitogenome), and the boundaries of each bin were found (Supplementary Fig. S4a). PCR amplification and appearance of target boundaries (Supplementary Fig. S4b, Supplementary information 1) further advocate high reliability of *O. glaberrima* mitogenome.

**Table 1:** Basic genetic index of the mitochondrial genomes in BIL lines

Lines	Genome size (bp)	GC content (%)	Gene region size (bp)	Non-gene region size (bp)	Scaffold number
*O. glaberrima*	402,174	44.0	71,174	331,000	2
P10	419,905	43.8	73,305	346,600	3
P88	414,702	43.7	73,230	341,472	2
P90	409,553	43.7	72,191	337,362	2
P91	441,199	43.7	74,840	366,359	3
P92	422,918	43.7	74,064	348,854	3

Likewise, the draft assembly of mitogenomes of five BILs was constructed (Supplementary Table S2, S3), and each mitogenome contained 2–3 scaffolds, with nearly stable GC content (~ 43%) as the maternal line, and genome size varied from 409,553 to 441,199 bp, a little larger than the *O. glaberrima* (Table 1). The mitogenome gene region is relatively more stable than the non-gene region, and the length of the gene region increased from 1017 to 3666 bp, whereas the non-gene region increased from 6362 to 35,359 bp (Table 1), implying the non-gene region as the main cause for the expansion of BIL mitogenomes. Prediction analysis revealed that each BIL mitogenome contained identical sets of protein and rRNA genes as the *O. glaberrima* did, including 35 protein genes, 3 rRNA genes, and 18 tRNA genes (Supplementary Fig. S5).

### Mitogenome of BILs showed distinctive structural variation

**Fig. 2:**
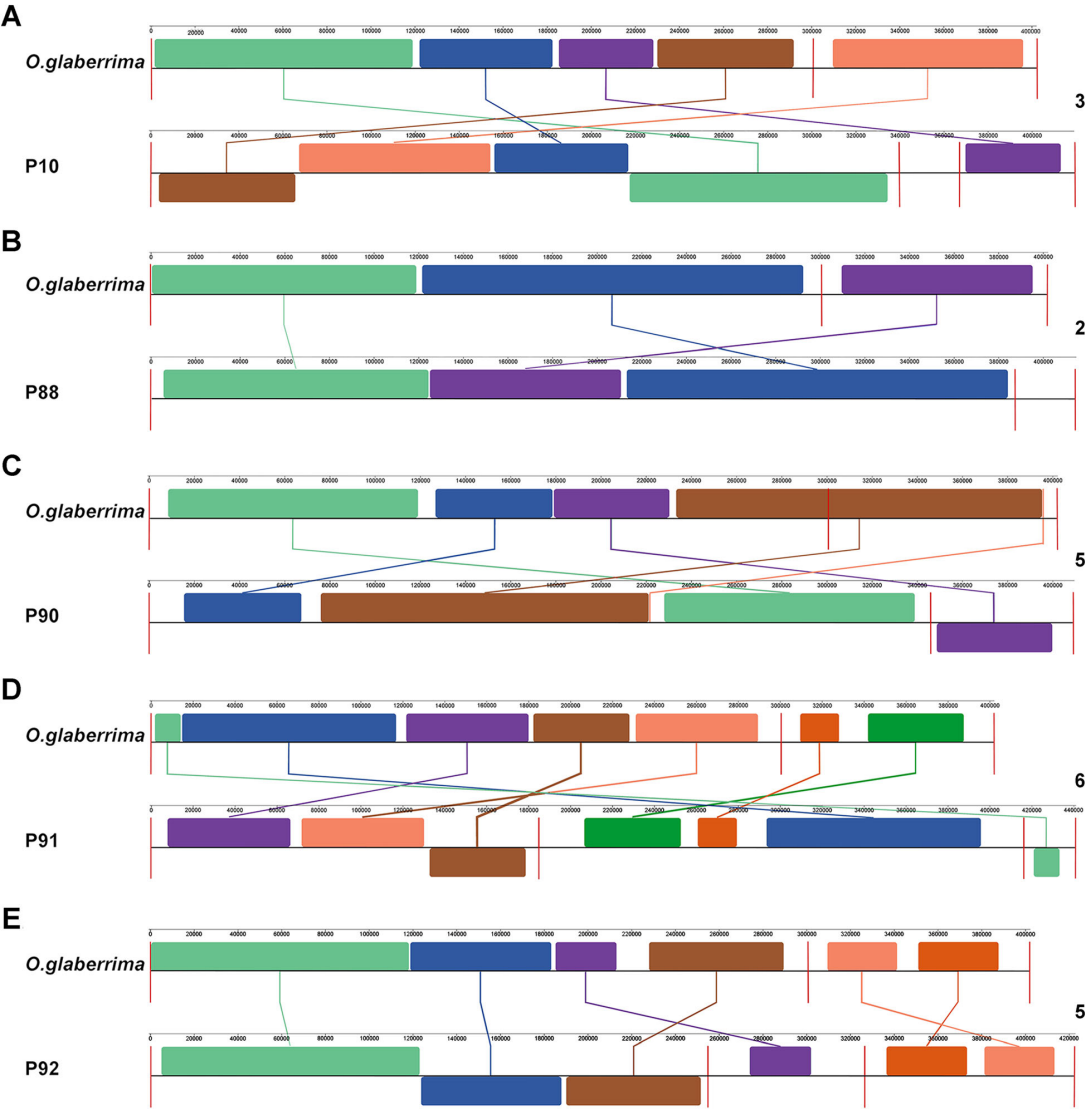
Collinearity analysis of the mitogenomic blocks between *O. glaberrima* and BIL lines. **a ~ e**, represent alignment of the mtDNA blocks of *O. glaberrima* to P10, P88, P90, P91 and P92, respectively. Red lines in each panel represent “boundaries” between scaffolds. The numbers on the right side of each panel represent the rearrangement events

When the BIL mitogenomes were aligned to the *O. glaberrima*, the pairwise genome alignment showed that there were 5, 3, 6, 7 and 6 homologous segments in P10, P88, P90, P91, and P92, respectively, being mapped to *O. glaberrima* (Fig. 2). These findings are in agreement with the 3, 2, 5, 6 and 5 rearrangement events in P10, P88, P90, P91, and P92, respectively as detected by GRIMM [20]. Comprehensively, the rearrangement events included 1 reversal and 2 translocations in P10, 1 translocation and 1 fusion in P88, 4 reversals and 1 translocation in P90, 2 reversals, 3 translocations and 1 fission in P91, and 2 reversals, 2 translocations and 1 fission in P92. The varied and inconsistent rearrangement pattern in mitogenomes signifies their strong diversity among the BILs.

Although alignment showed that the mitochondrial gene content is the same between BILs and maternal line, there were 15 genes with increased copy-number in at least one BIL except for the *trnP (TGG)* that showed a coinciding rise and fall in copy-number in different lines (Supplementary Table S4). Additionally, there were 20 new formed gene clusters in the BIL mitogenomes (Supplementary Table S6) due to the rearrangement. For example, the gene cluster *trnE (TTC)-rrnL-rrn5-rrnS* in *O. glaberrima* was converted into *trnE (TTC)-rrnL-atp6-nad5* in P10 and P92. Similar changes were also identified in P10, P91, and P92 (Supplementary Table S6), indicating that the mitogenome structure is globally transformed in the BIL population. Further analysis also confirmed that all mitogenomic variations in BILs were ascended from the maternal mitogenome itself, because any 93–11-specific mtDNA fragment (Supplementary Table S7) could not be detected in BIL mitogenomes.

### Homologous recombination and non-homologous end joining (NHEJ) equally contributed to mitogenome variation

It is well documented that rearrangements in angiosperm mitogenomes are largely mediated by repeat sequences, and their homologous recombination makes mitogenome a multipartite structure [21]. Accordingly, we analyzed the type and distribution of the repeats ≥50 bp which are regarded as the main contributor to mitogenomic rearrangement in plants. In total, 118 repeats including 15 large repeats (> 1000 bp) and 99 intermediate repeats (≥50 and ≤ 1000 bp) were identified in *O. glaberrima* mitogenome by BLASTALL, covering 24.14% of the mitogenome (Supplementary Table S9). Based on the repeats and flanking sequences, all the repeats were classified into 33 types, comprising of 30 two-copy repeats, 2 three-copy repeats, and 1 ten-copy repeat (Supplementary Table S10).

**Fig. 3:**
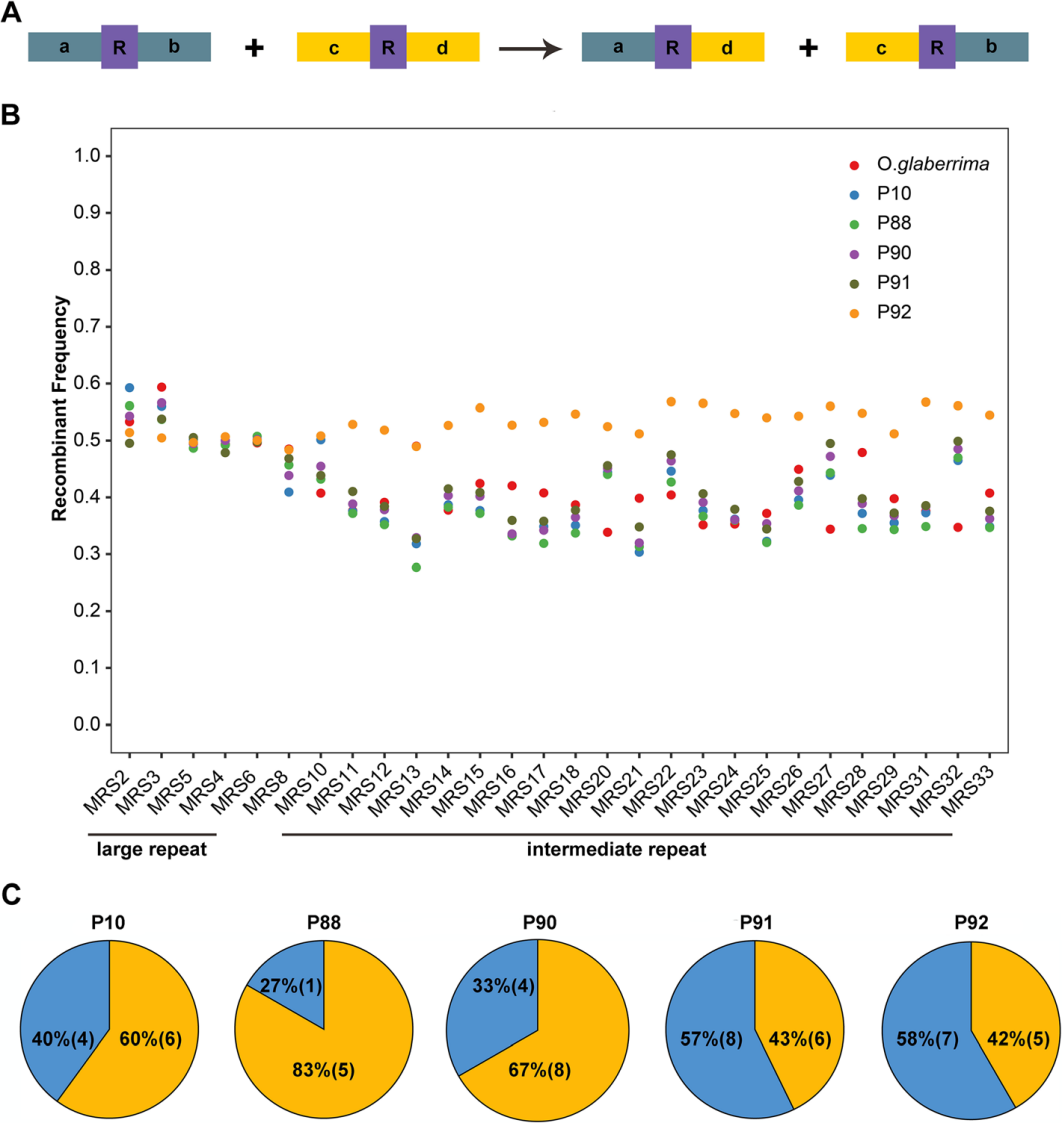
Recombination frequency in mitogenomes of BIL lines. **a**. Diagram of the recombination manners mediated by two-copy repeats. **b**. Recombination rate of all repeats with two copies in BIL mitogenomes. **c**, Frequency of the repeat mediated and non-repeat mediated recombination in mitochondria of BIL lines. The yellow represents repeat mediated recombination and the blue represents non-repeat mediated recombination, the number after each Mauve alignment represents the number of rearrangements inferred

Then, the 28 two-copy mitochondrial repeats with detectable flanking sequences were selected for recombination analysis. Since the two-copy repeats generate only two products after recombination (Fig. 3a), it makes them convenient for detection [22]. When the reads of the 20 Kb PacBio sequencing library to the mitochondrial repeats were mapped, huge variations in the repeat configurations (repeat + flanking sequence) of the same repeat were detected among BILs. This was illustrated by reduced MRS10-cd to 30.1% in P88 and increased MRS5-cd to 372.5% in P92 relative to the maternal line (Supplementary Fig. S6). It infers that homologous recombination (repeat-mediated) largely occurred in the BIL (Fig. 3b), as further verified by qPCR analysis that both MRS8-cb and MRS8-ad in P88 mitogenome were 100 times more than that in *O. glaberrima* (Supplementary Fig. S7).

Generally, the recombination rate in P91 and P92 is higher than the others. The intermediate repeat-mediated recombination became dominant in the BIL mitogenomes, and the range of variation caused by intermediate repeats was apparently wider than the large repeats (Fig. 3b), reflecting a key role of intermediate repeats in regulating mitogenome structure. Further analysis also showed that the repeat-mediated homologous recombination can lead to the change in gene order (Supplementary Fig. S8), as shown by GJ01 in P10 and P91, consistent with gene cluster change in BIL lines (Supplementary Table S6).

Interestingly, when we detect if repeats are overrepresented at rearrangement boundaries, the overlapping rate ranges from 42 to 83% in different BILs (Fig. 3c; Supplementary Table S8), stating that nearly half of the rearrangement events are caused by non-homologous end joining (NHEJ), which also plays an important role in determining the mitogenome structure. This is consistent with the increase in copy-number of 15 mitochondrial genes in BILs (Supplementary Table S4), as the NHEJ is usually related to mitochondrial gene copy number in plants as well as animals [23, 24]. Further analysis of the recombination patterns also showed that one-third of the reversal, half of the translocation, and most of the fission events were involved in non-homologous recombination (Supplementary Table S5). These results confer the non-homologous recombination as one of the major driving forces for mitogenome rearrangement.

### Genomic variations alter mitochondrial gene expressional profile in BILs

**Fig. 4:**
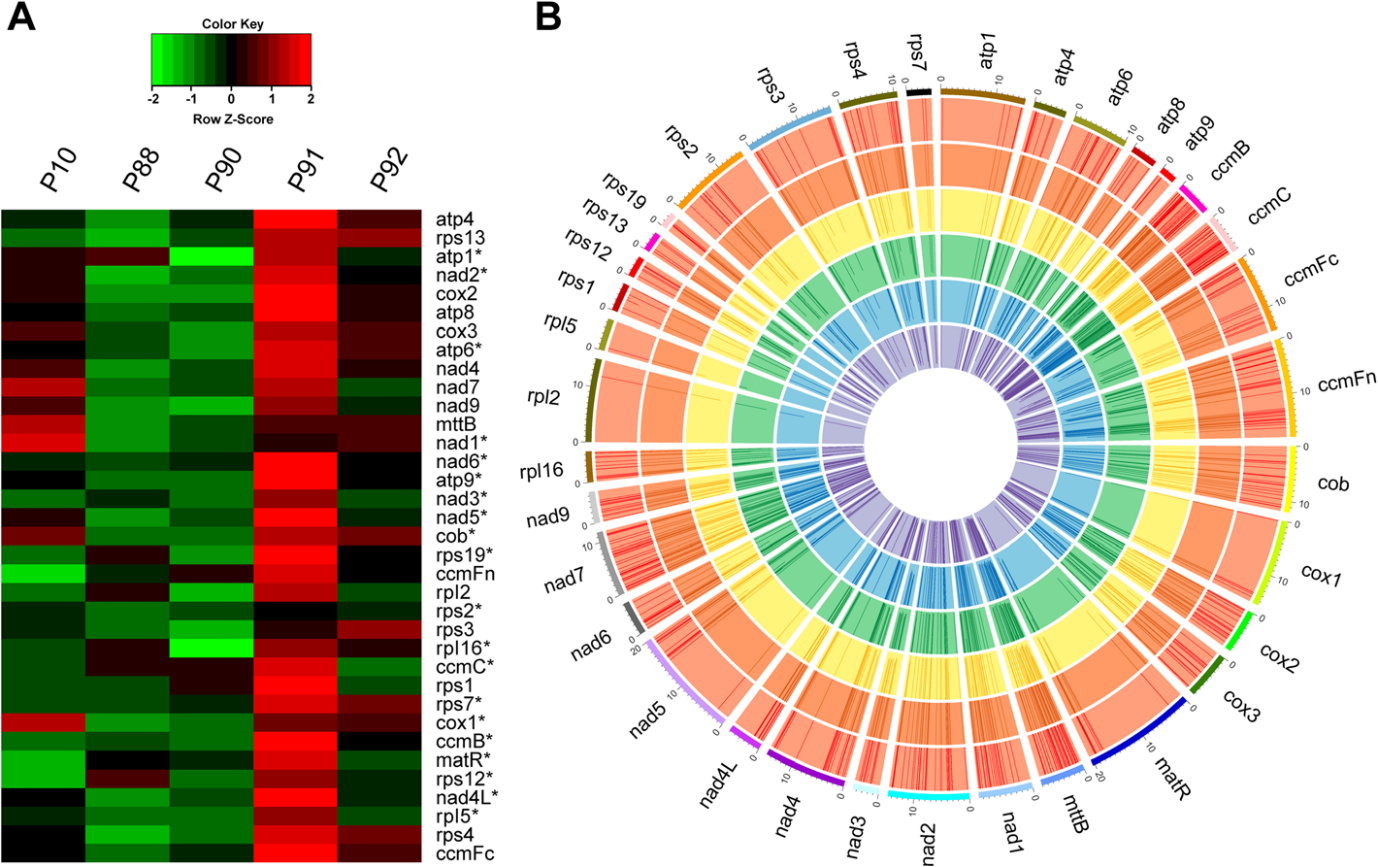
Relative expression and RNA-editing rate and sites of mitochondrial genes in maternal parent and BIL lines. **a**, Heatmap of the expression profile of mitochondrial genes. Asterisk represents the gene which is near within 2 Kb of repeat. **b**, Circle map for RNA-editing rate and sites of mitochondrial genes. From inside to outside, the circle in order represents *O. glaberrima*, P10, P88, P90, P91 and P92, respectively. The line length in the circles represents the RNA editing rate

Although the recombination mostly occurred in the non-gene region, about 57.1% coding genes were located within 2 Kb upstream or downstream of repeat sequences (Supplementary Table S9), suggesting the role of mtDNA recombination in promoter structure and activity as reported in the family of *Silene vulgaris* [11]. Therefore, transcriptome analysis combined with qPCR was carried out to evaluate the expression profile of mitochondrial genes. Transcriptome analysis showed that the expression level of functional mitochondrial genes among the BILs ranged from 0.27 to 4.16 times of the *O. glaberrima* (Fig. 4a). Of which, the expression of *atp9*, *rps19* and *rps2* reduced to over 50% than *O. glaberrima*, whereas, the expression of 15 genes including *atp9*, *ccmB*, *ccmC*, *ccmFc*, *cox1*, *nad1*, *nad4L*, *nad6*, *rpl16*, *rpl5*, *rps1*, *rps12*, *rps3*, *rps4,* and *rps7* was increased over 50% than *O. glaberrima* (Fig. 4a). Further correlation studies between gene expression and repeats showed that all the genes with over 50% down-regulation and 73% of the genes with over 50% up-regulation were all adjacent to the repeat within 2 Kb. The qPCR analysis also showed that expression levels were positively correlated with their gene contents, up to 2 Kb proximal repeats (Supplementary Fig. S9), inferring an important role of repeats in regulating mitochondrial gene expression. In general, expression of the genes in P91 is higher than that of P92, followed by P10, P88, and P90, respectively (Fig. 4a); the exact mechanism is yet to be ascertained. This reflects the complex nature of expression regulation in mitochondria.

### The mitochondrial genes in BILs showed different RNA-editing pattern

RNA-editing plays a key role in coordinating mito-nuclear interaction [25]. Mitogenomic structural variation serves as a dynamic means for mitochondria to deal with the cytonuclear interaction. Therefore, the RNA-editing site and rate of the protein-coding genes with reads detected ≥50 times were assayed in each mitogenome (Supplementary information 2). In general, a total of 525 C-to-U and 4 U-to-C editing sites involved in 35 mitochondrial genes were identified in the coding sequences (Supplementary Table S11). Notably, about half of the editing sites were located at the second codon, and 459 editing sites were related to amino acid change (Supplementary Table S11) in 35 genes (Supplementary information 1). With reference to the maternal line, the rise and fall of the RNA-editing rate in the same site were frequently observed among BILs (Fig. 4b). This is enormously demonstrated by the sites of *mttB*-381 having an editing rate of 35.5% in *O. glaberrima*, but 50.5% in P10 and only 21.7% in P90 (Fig. 4b), suggesting the dynamic nature of RNA-editing even for the same site in different lines.

Currently, a total of 339 mitochondrial RNA-editing sites are known in the coding sequence of rice (https://www.ncbi.nlm.nih.gov/). When these RNA editing sites were cross-referenced with the Nipponbare, about 143 mitochondrial RNA editing sites were found to be unique (Table 2). In order to test the accuracy of the bioinformatics analysis, three editing sites (*cox2*–144, *mttB*-354 and *nad7*–1110) with a large disturbance were selected for re-sequencing, and all of them showed similar editing pattern as anticipated by bioinformatics analysis (Supplementary Table S12), signifying their consistency.

**Table 2:** Comparison of the RNA editing in mitogenome of the BIL lines

Lines	Edited genes	Edited sites	Newly detected sites	Editing-rate changed sites
*O*. *glaberrima*	35	501	132	–
P10	32	465	120	111
P88	35	517	135	95
P90	35	523	135	105
P91	35	524	137	109
P92	35	516	133	91

Notes: New sites, newly detected RNA-editing sites. Site of editing rate changed: the editing rate increase or decrease more than 10%

## Discussion

This is the first study on the mitogenome variation of maternal inheritance in plants that uncovers a novel mechanism of the organelle genome corresponding to adapt a reshuffled heterogenic nuclear genome. The transmitted mitogenome revealed a global rearrangement (mostly occurring as reversal) through repeat-mediated recombination and non-homologous end joining (NHEJ) (Fig. 2f) and extensive alterations (including gene copy-number, expression, and substantial RNA-editing sites) that occurred in the progenies. These findings indicate that the inheritance of mitogenome across the descendent plants is dynamic and different progenies hold varied mitotypes. Surprisingly, expression levels were not always positively related to copy-number in the tested BIL lines, which reflects a complicated mechanism of mitochondria to cope with the nuclear-cytoplasmic interaction in plants.

### Wide crossing alters mitotypes of progenies

It is well documented that cytoplasmic inheritance in rice and most of the other higher plants is typically due to maternal inheritance. However, increasing reports of the complicated mitotypes in rice seems compromising this opinion [19, 26]. In the present research, paternal mtDNA was not transmitted to the progenies (Supplementary Table S7), but the mitotypes in BIL lines were strongly differentiated (Fig. 2). MtDNA clustering analysis clarified that the paternal-backcrossed BIL population was apparently much more differentiated than that of the maternal-backcrossed BIL population (Fig. 1). Even in the same BIL population, the lines carrying more DNA fragments from the paternal line showed stronger mtDNA differentiation than the others. These findings suggest that the heterogenic nuclear genome could promote variation in the mitogenomes so as to concert the nuclear-mitogenomic interaction, keeping normal development of the BIL lines (Supplementary Fig. S2A).

Common wild rice is the direct ancestor of cultivars. The cultivars and wild rice with AA-genome can generate hybrids through outcrossing to some extent. Naturally, cultivars are frequently overlapped with habitant of wild rice, especially *O. rufipogon* in Asia, which inevitably lead to genetic drift [27]. Meanwhile, wild rice with AA-genome is the most important gene pool for rice improvement. The vast number of favorable genes such as *Gn8.1* for grain yield, *Bph14* and *Bph15* for brown plant hopper [28], *Xa21* and *Xa23* for white leaf blight [29] have been transferred to cultivars by crossing with wild rice. The natural and artificial out-crossing brings many of the genetic exchanges between wild rice and cultivars at genome level [30]. As exemplified by the cytoplasmic male sterility, a detrimental mitochondria-nuclear interaction caused by interspecific hybridization in the plant system like rice [31], cotton [32], rape seed [33], sorghum [34] etc., and harmonious nuclear-cytoplasmic interaction play a critical role in maintaining plant normal growth, fertility, survival, and even speciation [35]. In the wild or artificial hybridization, frequent introgression from wild rice not only reshuffles the nuclear genome but also dynamically sorts the mitogenomes through rearrangement. It helps cope with the introgressed nuclear elements to maintain stability in the species population. Thus, it will drive the variation and evolution of the mitochondrial genomes.

### Recombination of mitogenome is far from repeat sequences

Plant has a complex and dynamic mitogenome configuration [36], which prefer to undergo active inter- and intramolecular DNA exchange [37]. For mitogenome, recombination is the main contributor of its genetic variation, leading to stoichiometric variation in copy number, intragenomic rearrangements, and heteroplasmic molecular populations [13]. Genomic rearrangement occurs through homologous recombination and non-homologous end-joining (NHEJ), where homologous recombination is mainly subjected to active repeat sequences [37]. Plant mitogenomes are characterized by abundant repeat sequences of different sizes and number. Previous reports reveal that the recombination-active large repeats are responsible for the highly variable structure of plant mtDNA [38]. In this study, the number of intermediate repeats was at least six times of the large repeats (Supplementary Table S9). Furthermore, the intermediate repeats involved in recombination frequency were apparently more than that of the large repeats in *O. glaberrima* as well as BILs (Fig. 3), indicating that majority of the mtDNA recombination are owing to intermediate repeats.

Unlike the significant co-occurrence of homologous region boundaries and repeats in other plants [39–41], there was at least 27% of non-homologous recombination being detected in different BIL mitogenomes (Supplementary Fig. S3C). Especially for P92, only 42% homologous region boundaries corresponded to the repeat sequence, while the majority of the mtDNA rearrangement was caused through NHEJ. This fact was underestimated in the previous studies [21], reflecting that NHEJ plays an equivalent role as that of homologous recombination. Overall, nine reversal, nine translocation, one fusion, and two fission rearrangement events were detected in BILs. Out of which, only 72% reversal sites, 50% translocation sites, and 25% fission sites were generated by homologous recombination events (Supplementary Table S5). Previous studies in plants and humans have confirmed that microhomology-mediated NHEJ involves the occurrence of copy-number variation of genes in mitogenomes [23, 24]. In this study, 15 genes were identified with increased copy-number (Supplementary Table S4), which corresponds well with the global NHEJ in BIL lines [42]. The findings indicate that both of the repeat-mediated recombination and non-homologous recombination contribute almost equal importantly to the differentiation of mitogenomes in rice.

In the *O. glaberrima*/93–11//93–11 BIL population, the molecular diversity was obviously more than that of *O. glaberrima*/93–11//*O. glaberrima* (Fig. 1), reflecting high consistency of mitogenome in most lines of *O. glaberrima*/93–11//*O. glaberrima* population. Although the mitochondrial genome is inherited independently to some extent, the DNA replication, RNA transcription, protein translation, and ATP production cannot work without precise cytonuclear co-adaptation [35]. In the five tested BIL lines, the mitogenomes were differentiated at different levels (Fig. 2). The BIL line P91 bearing more 93–11 genomic fragments showed stronger mtDNA rearrangement than the BIL P10 which bears less 93–11 nuclear fragment (Supplementary Fig. S3), even though the relationship was not completely positive correlated. The probable justification is that the mitogenome needs to reprogram to accommodate the reshuffled nuclear genome when the nuclear genome is shocked by alien genome.

### Dosage effect and RNA-editing play predominant roles in plant Mito-nuclear interaction

Natural distant hybridization is the main driving force for species formation [43] and interspecies hybridization usually leads to cytoplasmic male sterility because of species isolation [44–46]. The artificial wide crossing is a process to mimic the evolution in nature to select elite descendent with favorable agronomic traits. Here, the sterile lines in the BIL population were deleted after artificial selection (Supplementary Fig. S2). The analysis of the mitogenomes provided a medium to understand the evolutionary trends and adapt the nuclear genome reprogramming. Strikingly, global mtDNA rearrangement changed 15 gene copy-numbers (Table 1), and at least 20 gene clusters in the mitogenome (Fig. 2; Supplementary Table S6). These changes extensively modify the expression profile of mitochondria in rice (Fig. 4). The results demonstrated that the dosage effect plays an important role in regulating mito-nuclear interaction, although the exact mechanism needs to be investigated further.

Increasing reports have evidenced that RNA-editing is involved in regulating plant growth and development [47, 48]. It is considered that RNA editing implicates in the rescue of the organelle dysfunction caused by genetic, physiological or environmental factors [25]. Interestingly, 529 mitogenomic RNA editing sites were identified in this research, of which, 143 editing sites were detected for the first time in rice (Table 2). It is worth noting that about 21.0% of the editing sites showed disturbance in their editing rates among BIL lines in relation to the maternal lines (Fig. 4b). The difference in the RNA editing site and the rate in BIL lines are strongly in agreement with the nuclear heterogeneity. To some extent, it reflects the co-adaption of the mitochondria and nuclear genomes, because the PPR protein encoded by the nuclear genes controls the RNA editing. It is a well-known fact that the PPR gene family is mainly responsible for the RNA-editing and rapidly gets expanded as well as diversified in the higher plants during the course of evolution [49, 50]. Considering the fact that animals have a few PPR members, lesser DNA rearrangement, and low RNA editing [37], it is proposed that mitochondria are functionally specified for energy production, which is a crucial role in organism development. In order to maintain a harmonious nuclear-cytoplasmic interaction, the PPR was actively evolved in plants to modify or trigger the organelle transcripts for adapting the mutated subunit of complex proteins encoded by nuclear genome.

## Conclusion

In the present study, it was found that BIL mitogenomes transmitted from the maternal line were globally reprogrammed through DNA recombination in multiple ways. The global rearrangement changed genome structure, organization, and gene copies that altered the expression level of genes. Meanwhile, the RNA editing site and editing rate were also extensively changed, along with the dosage effect that triggers the interaction between nuclear and mitochondria to a concerted state.

## Methods

### Plant materials and culture

The 3 K rice lines were provided by Dr. Binying Fu, institute of crop science, Chinese academy of agricultural sciences, Beijing 100,081, China. *O. glaberrima* (ICR650) was provided by international rice research institute, Philippines. 93–11 was provided by Yangzhou academy of agricultural sciences, Yangzhou 225,007, China. Two BC_2_F_12_ BIL populations (ICR650/93–11//93–11 and ICR650/93–11//ICR650), which were built by us, derived from a wide cross of *O. glaberrima* × *O. sativa* (ICR650 × 93–11) and the maternal line ICR650 were used in this research. All the plants were planted in the camp of Wuhan University with standard management.

### Detection of mitochondrial genotypes

Total genomic DNA was extracted from 2-week-old rice seedlings by the CTAB method. PCR amplification was performed using mitotype-specific molecular markers (MSS3, MSS5, MSS7, MSS8, MSS12, MSS13, MSS20, MSS21, MSS25, MSS27, MSS28, MSS29, MSS32, MSS33, MSS34, MSS37, MSS38, MSS39, MSS41, MSS42, MSS43, MSS44, MSS46, MSS47, MSS50, MSS51, MSS52, MSS53, MSS54, MSS55, MSS56 and MSS57) [19] in a 10 μL final reaction mixture containing 3.7 μL of distilled water, 5 μL 2 × Taq Plus Master Mix II (Vazyme, China), 0.4 μL of primer (10 pmole/μL) and 0.5 μL of genomic DNA (50 ng). All the reactions were set at an initial denaturation step (95 °C for 5 min), 30 cycles of denaturation (95 °C for 30 s), annealing (50 ~ 60 °C for 30 s), and extension (72 °C for 20 s ~ 70 s, depending on the size of the amplified fragment) and final extension (72 °C for 5 min). Amplification products were evaluated by running on 1.5% agarose gel. Each band produced by PCR was treated as a unit character and scored as a binary code (1/0). The phylogenetic tree was constructed by the neighbor-joining method, implemented in DPS (Data Processing System) (v17.10) [51] and MEGA X [52]. The landscaping appearance of the tree was obtained by FigTree v1.4.3 (http://tree.bio.ed.ac.uk/software/figtree/).

### Bin map construction

Total genomic DNA extracted from 2-week-old rice seedlings was sent to BENAGEN for sequencing on the Illumina HiSeq/MiSeq platform with the 400 bp paired-end library. Adapter and low-quality sequencing reads were trimmed by FastQC. Nipponbare genome (IRGSP-1.0) was downloaded from EnsemblPlants as a reference and all clean data were compared by BWA. GATK (Analysis Tools for Next-generation DNA Sequencers) v3.7.0 was used to call all SNPs. The maps of BILs were aligned and compared to their genotypes for a 15 SNP interval. Adjacent 15 SNP intervals across all BILs with the same genotype were combined into a recombination bin.

### Mitochondrial genome sequencing, assembly, and annotation

Rice mitochondria were isolated from callus by differential centrifugation method followed by continuous Percoll gradients as described by Heazlewood et al. [53]. The DNA was extracted from the rice mitochondria by CTAB and sequenced by NextOmics (Wuhan, China) on an Illumina HiSeq Xten machine with the 400 bp paired-end library, and on a PacBio RSII machine with 20 kb paired-end library.

The raw reads from PacBio RSII longer than 26 Kb were used as seed reads. The reads shorter than 26 Kb were corrected by the RS_PreAssembler.1 protocol with default settings from the Pacific Biosciences SMRT analysis (v2.3.0) software package. In addition, the raw reads from Illumina HiSeq Xten were also used to correct the genome by pilon (−-changes --vcf --fix bases --threads 5 --mindepth 10). Mitochondrial genomes were annotated using MITOFY [54]. The tRNA genes were searched using tRNAscan-SE [55]. Finally, the genome maps were drawn by OGDRAW [56].

### 93–11 special sequence detection

Mitochondrial genome (mitogenome) of *O. glaberrima* was aligned against 93–11 (NC_007886) using BLASTN [57] with a word size of 20 nucleotides and expectation value of 1. Specific sequences in the 93–11 mitogenome were extracted.

### Genome rearreangement

To identify mitochondrial synteny blocks between the pairs of representative BIL lines, all mitochondrial genomes were aligned using Mauve version 2.3.1 [58] with an LCB cutoff of 500, respectively. Pairwise rearrangement distances in terms of minimum number of rearrangements were inferred using GRIMM [20].

### Repeats and repeat-mediated recombination

Repeats were detected using BLASTN to search *O. glaberrima* mitogenome against itself with a word size of 50 nucleotides and an expectation value of 1. Each repeat copy was numbered and distinguished by the flanking sequences [22]. The repeat sequences having only two copies were shorter than 1 Kb and not at the end of the scaffold were chosen to design primers (Supplementary information 1) by Primer 5 [59] for detection.

The frequency of repeat-mediated recombination was evaluated for all repeats in mitogenomes that had only two copies and were not at ends of scaffold using procedures described by Mower et al. [60]. The parameters and calculation methods reported by Guo et al. [61] were used for our analysis.

The content of one repeat configuration is equal to the mapping read pair number divided by all mapping read pairs of repeat configurations. The relative content of one repeat configuration of BIL lines is equal to the content of repeat configuration in BIL lines divided by the content of repeat configuration in *O. glaberrima*.

### Adjacency of rearrangement breakpoints and repeats

HRBs (homologous-region boundaries) verified by pairwise Mauve alignments were considered as agencies for rearrangement sites following the method of Cole, et al. [21]. In brief, HRBs, which were at the extremity of scaffolds, were eliminated from our analyses. The proportion of HRBs that was close to repeats was equal to the number of HRBs within 50 bp of repeats divided by the total number of HRBs.

### RNA extraction and transcriptome analysis

Total RNA was isolated using TRIzol reagent (Invitrogen) from the 2-week-old rice seedlings. Ribosomal RNA content was reduced from total RNA following the method of Guo, et al. [62]. The Ribo-Zero™-treated organellar RNAs of all samples were sent to BENAGEN for 75 bp single-read sequencing on the Illumina HiSeq/MiSeq platform.

Adapter and low-quality bases of RNA sequencing data were trimmed according to Guo et al. [62]. Clean reads were mapped to mitochondrial genome by bowtie2 v2.3.4.1 [63] (−-no-mixed--no-discordant --gbar 1000 --end-to-end -k 200 -q -X 800). RSEM v1.2.25 [64] was used to calculate FPKM of the gene.

### Reverse transcription, fluorescence quantification, and RNA-editing verification

Approximately 4 μg of RNA was treated with DNase I (NEB) and cDNA was obtained by reverse transcription with SuperScriptII as described in the manufacturer’s instructions. Real-time quantitative PCR was performed using LightCycler 480 (Roche) and the SYBR Green I Master PCR kit (Roche).

### RNA-editing analysis

RNA-editing sites were identified following the method of Guo et al. [62]. The editing sites with a cover depth lesser than 50 were removed by SAM tools mpileup [65]. The cDNA containing the editing site was ligated into pMD18-T and the constructs were co-transformed into *E. coli*. For Sanger sequencing, 50 monoclonal constructs of every editing site were taken and the RNA editing rate was calculated.

## Supplementary information

**Additional file 1: Fig. S1.** Polymorphism of mitochondrial genomes in landraces was detected using 32 mitochondria-specific molecular markers. The green represents *Oryza sativa japonica*. The blue represents *Oryza sativa indica*.

**Additional file 2: Fig. S2.** Gross plant morphology of the maternal parent and BILs. **A.** Morphologies of the maternal line and BILs. Scale bars, 10 cm. **B.** Basic agronomic traits of BILs.

**Additional file 3: Fig. S3.** The bin map of five BIL lines. The green represents the genome fragment which from *O. glaberrima*. The red represents the genome fragment which from 93-11. The blue represents the genome fragment which is heterozygotic. 15 SNPs for a window.

**Additional file 4: Fig. S4.** Validation of the mitochondrial genome of *O. glaberrima*.. **A.** Comparison between Nipponbare mitochondrial genome and *O. glaberrima* mitochondrial genome. **B**. Verification of different structural sites.

**Additional file 5: Fig. S5.** Complete mitogenomes of the maternal parent *O. glaberrima* and five BIL lines.

**Additional file 6: Fig. S6.** Percentage of different configurations of mitochondrial two-copy repeats in BIL lines compared to the *O. glaberrima*.

**Additional file 7: Fig. S7.** Relative content of different configurations of some mitochondrial repeats in BIL lines compared to *O. glaberrima* by fluorescence quantification.

**Additional file 8: Fig. S8.** The change of gene order based on homologous recombination.

**Additional file 9: Fig. S9.** DNA content and RNA levels of some mitochondrial genes in BIL lines compared to *O. glaberrima* by fluorescence quantification. **A.** Relative DNA and RNA level to *O. glaberrima* of mitochondrial genes near repeat within 2 Kb. **B.** Relative DNA and RNA level to *O. glaberrima* of mitochondrial genes far away from repeat over 2 Kb.

**Additional file 10: Table S1.** Summary of sequencing nuclear genomes by Illumina. **Table S2.** Summary of sequencing mitogenomes by Illumina. **Table S3.** Summary of sequencing mitogenomes by PacBio. **Table S4.** Multi-copy genes in mitogenomes of the BIL lines. **Table S5.** Rearrangement types and recombination manners in BIL mitogenomes. **Table S6.** Variation of some representative gene clusters in mitogenome of BIL lines. **Table S7.** Special mitogenome sequences in 93–11. **Table S8.** Homologous region boundaries of BIL lines relative to *O. glaberrima*. **Table S9.** All repeats detected in *O. glaberrima* mitogenome. **Table S10.** Category of multi-copy repeats in *O. glaberrima* mitogenome. **Table S11.** RNA editing events detected in mitochondria of BIL lines. **Table S12.** Confirmation of RNA editing using Sanger sequencing

**Additional file 11 Supplementary information 1**

**Additional file 12 Supplementary information 2**

## Data Availability

The data of all DNA-Seq and RNA-Seq has being uploaded to the NCBI SRA database (http://www.ncbi.nlm.nih.gov/sra), with the accession number: PRJNA598996, Other data that supporting the conclusions of the article have been uploaded as additional files. Seeds are available upon request from the corresponding author.

## Abbreviations

BIL: Backcross inbred lines
Mitogenome: Mitochondrial genome
NHEJ: Non-homologous end joining
HRBs: Homologous-region boundaries
