# Supplementary figures and images for: Wide crossing diversify mitogenomes of rice
Source: BMC Plant Biol. 2020 Apr 15;20:159. doi: 10.1186/s12870-020-02380-w (PMC7160995; doi:10.1186/s12870-020-02380-w)

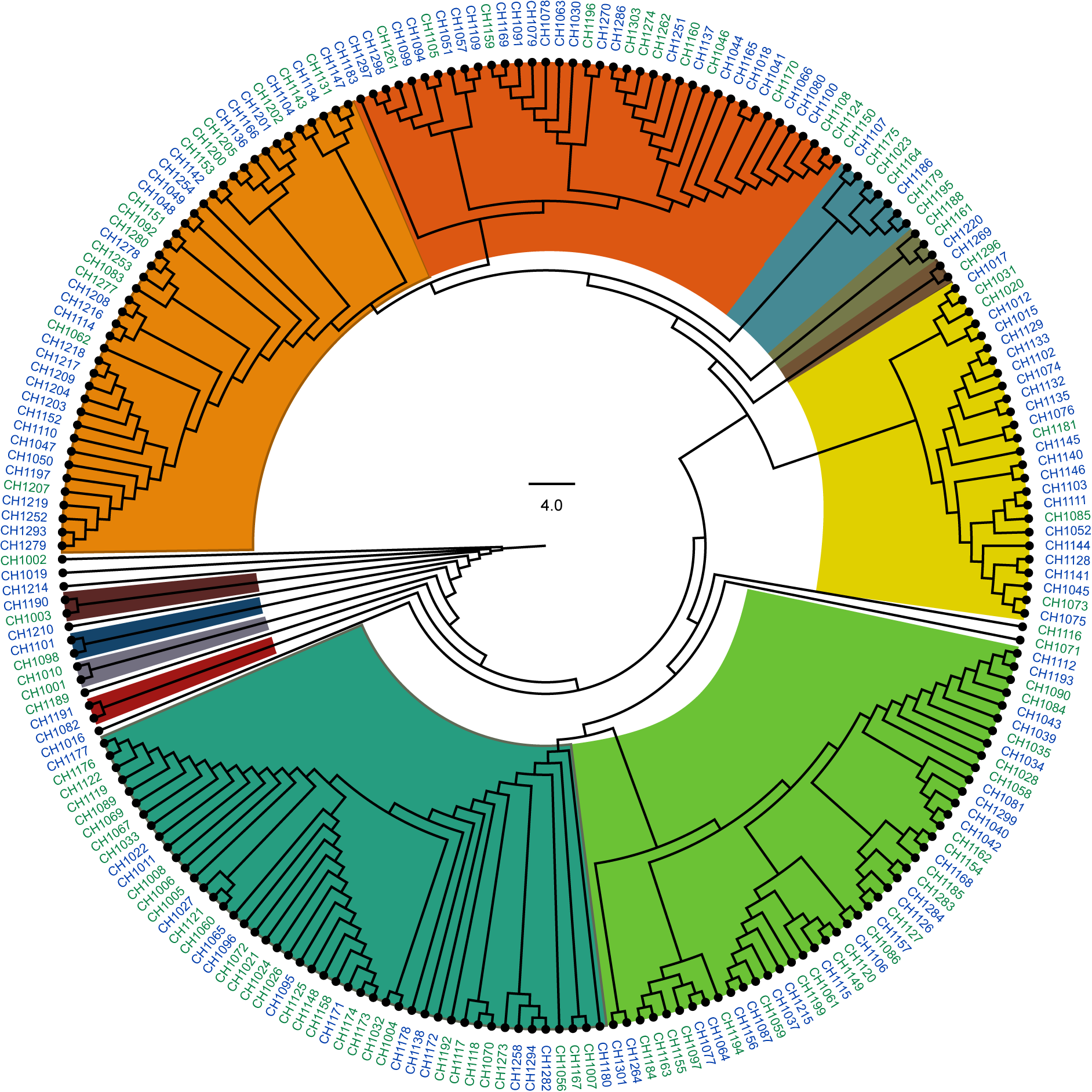

Supplement: Supplementary file 1 — Additional file 1: Fig. S1. Polymorphism of mitochondrial genomes in landraces was detected using 32 mitochondria-specific molecular markers. The green represents Oryza sativa japonica. The blue represents Oryza sativa indica. [file 12870_2020_2380_MOESM1_ESM.tif]

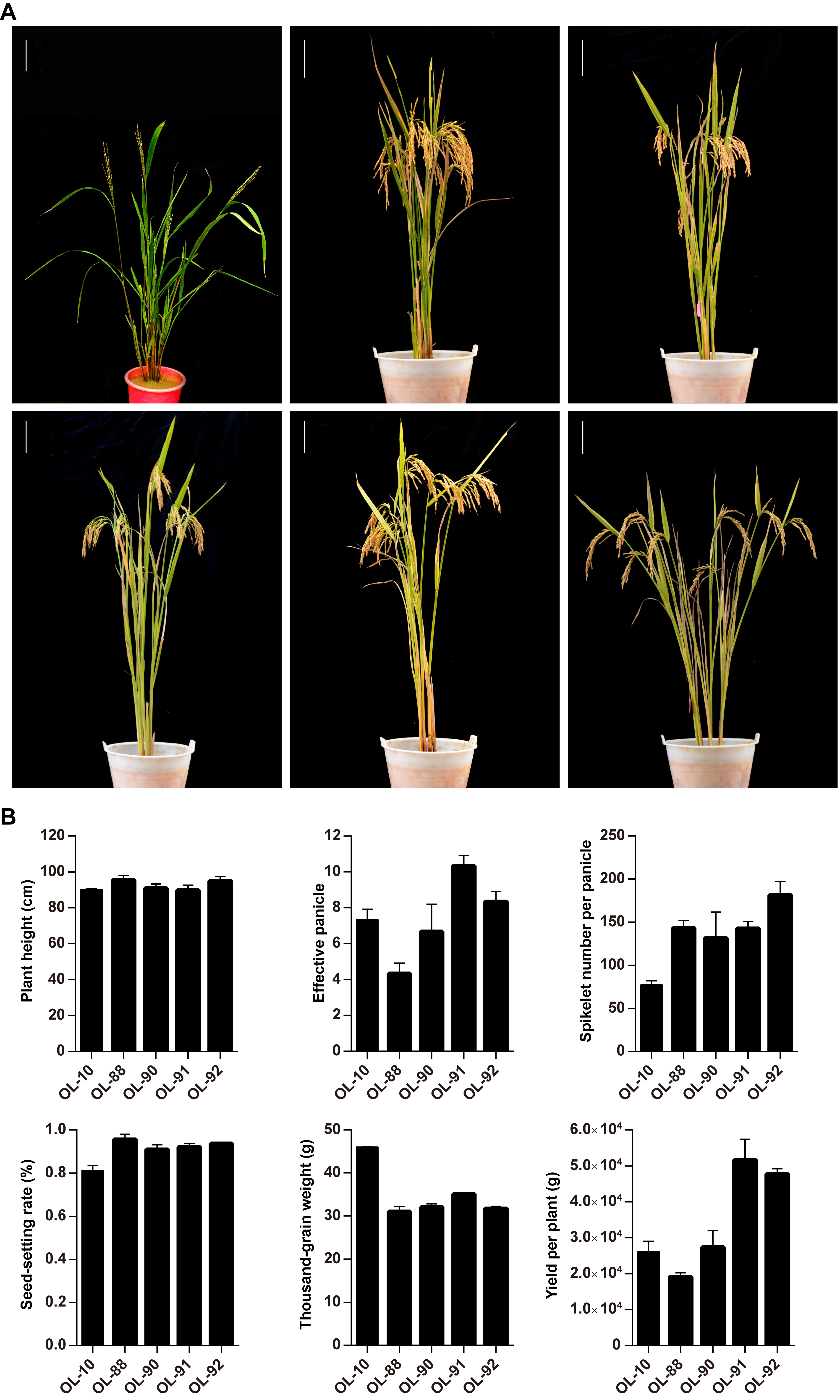

Supplement: Supplementary file 2 — Additional file 2: Fig. S2. Gross plant morphology of the maternal parent and BILs. A. Morphologies of the maternal line and BILs. Scale bars, 10 cm. B. Basic agronomic traits of BILs. [file 12870_2020_2380_MOESM2_ESM.tif]

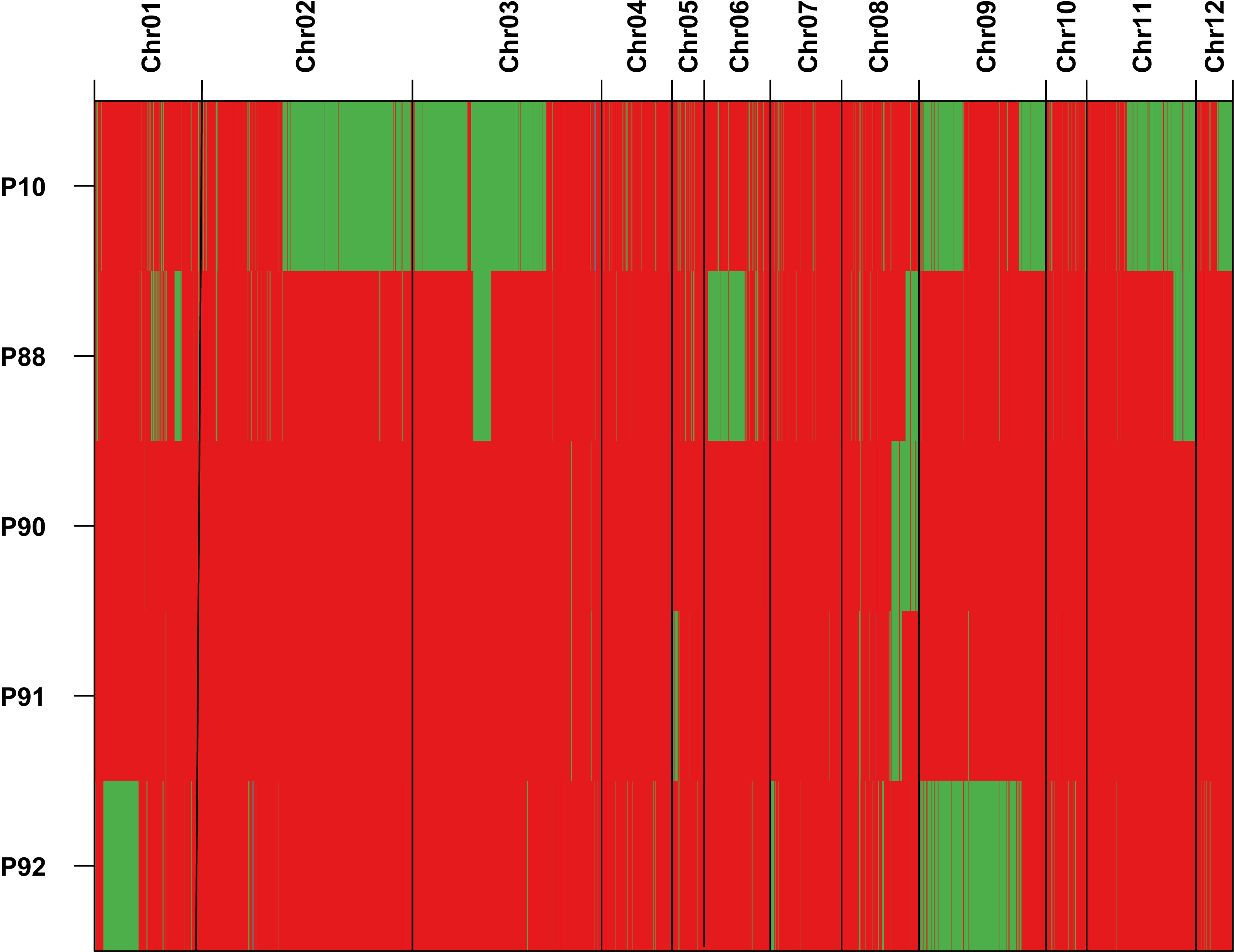

Supplement: Supplementary file 3 — Additional file 3: Fig. S3. The bin map of five BIL lines. The green represents the genome fragment which from O. glaberrima. The red represents the genome fragment which from 93-11. The blue represents the genome fragment which is heterozygotic. 15 SNPs for a window. [file 12870_2020_2380_MOESM3_ESM.tif]

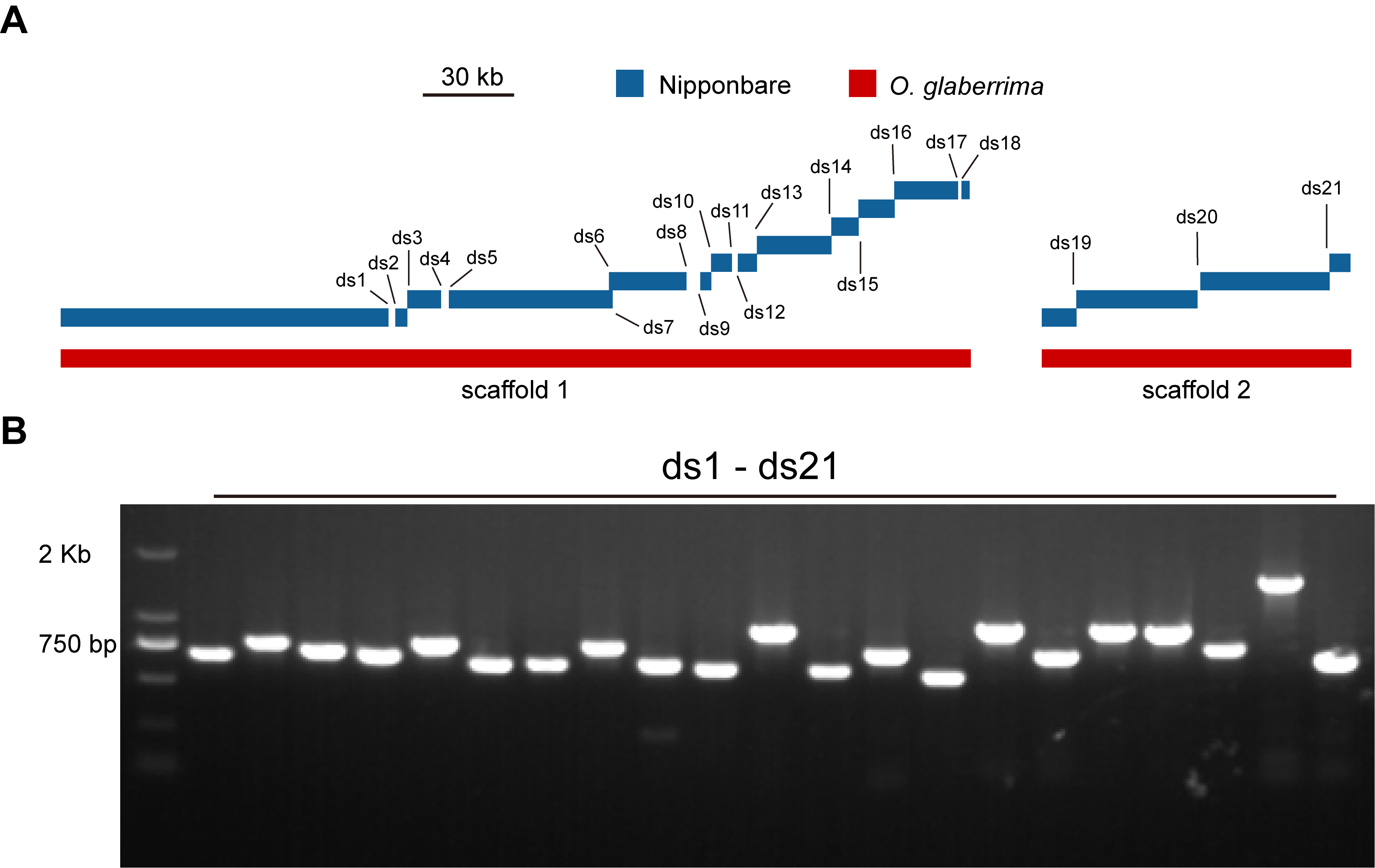

Supplement: Supplementary file 4 — Additional file 4: Fig. S4. Validation of the mitochondrial genome of O. glaberrima.. A. Comparison between Nipponbare mitochondrial genome and O. glaberrima mitochondrial genome. B. Verification of different structural sites. [file 12870_2020_2380_MOESM4_ESM.tif]

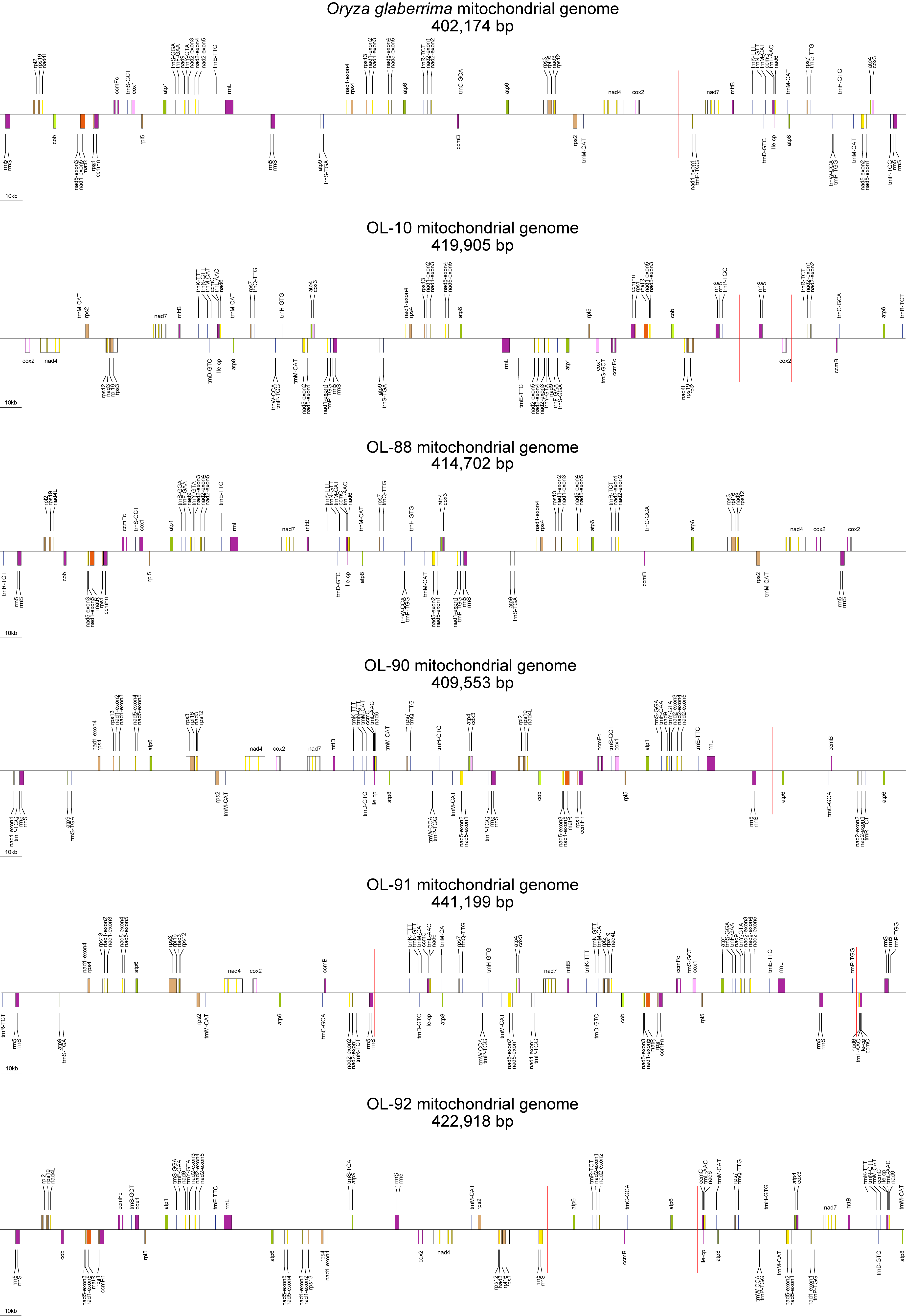

Supplement: Supplementary file 5 — Additional file 5: Fig. S5. Complete mitogenomes of the maternal parent O. glaberrima and five BIL lines. [file 12870_2020_2380_MOESM5_ESM.tif]

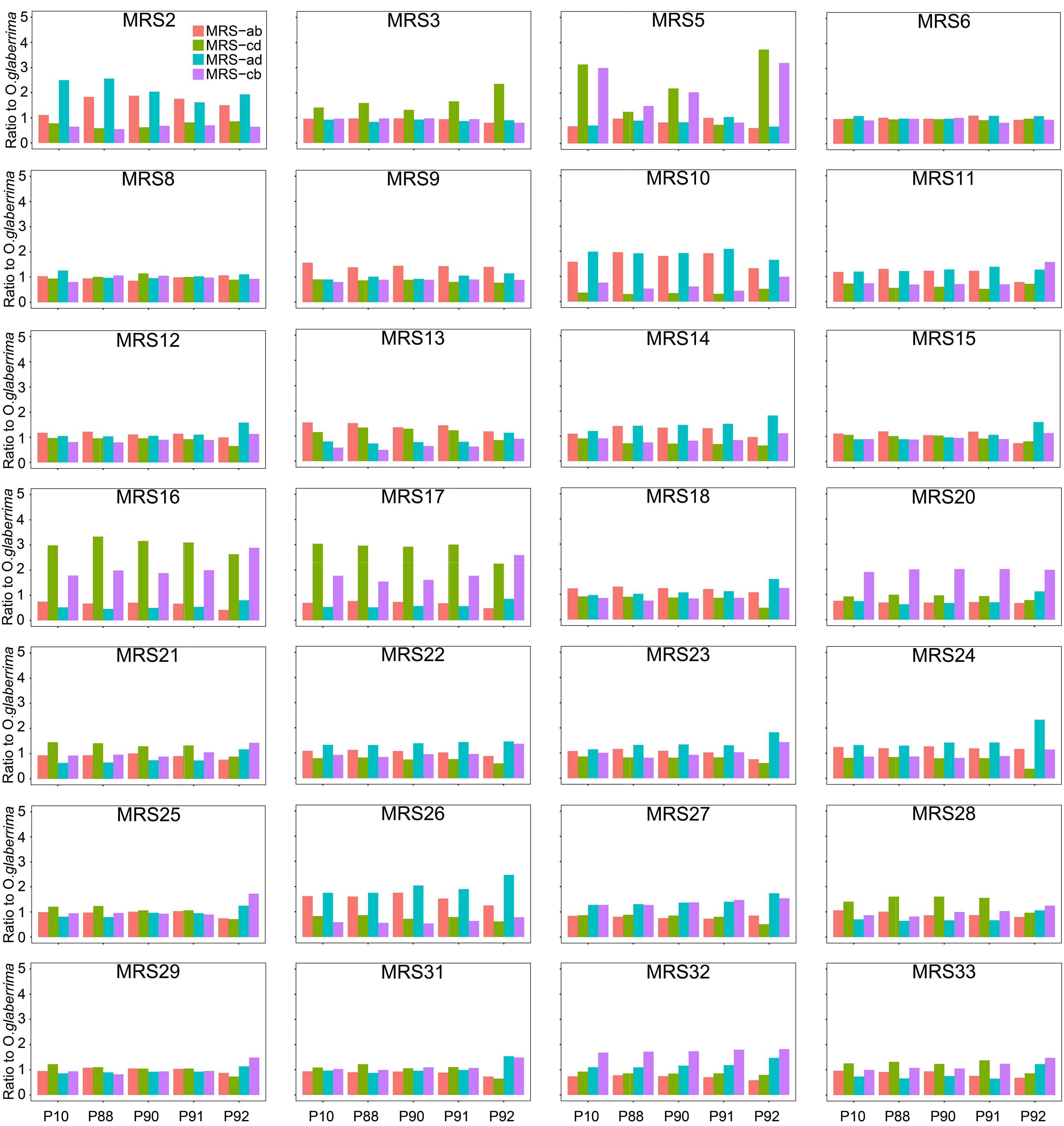

Supplement: Supplementary file 6 — Additional file 6: Fig. S6. Percentage of different configurations of mitochondrial two-copy repeats in BIL lines compared to the O. glaberrima. [file 12870_2020_2380_MOESM6_ESM.tif]

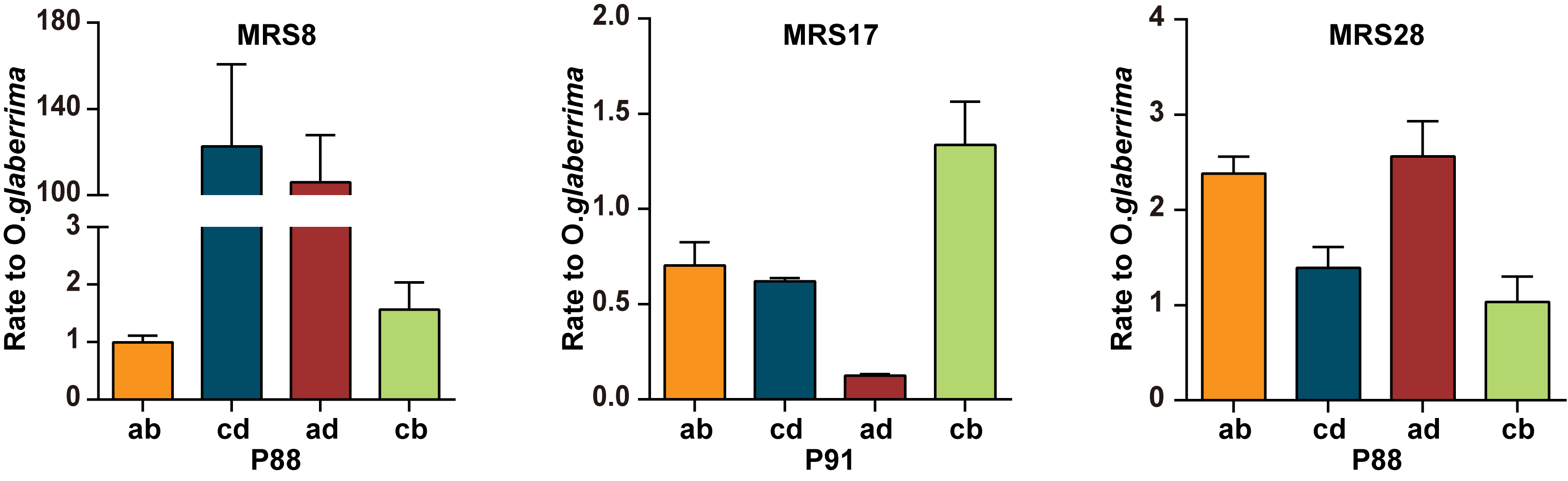

Supplement: Supplementary file 7 — Additional file 7: Fig. S7. Relative content of different configurations of some mitochondrial repeats in BIL lines compared to O. glaberrima by fluorescence quantification. [file 12870_2020_2380_MOESM7_ESM.tif]

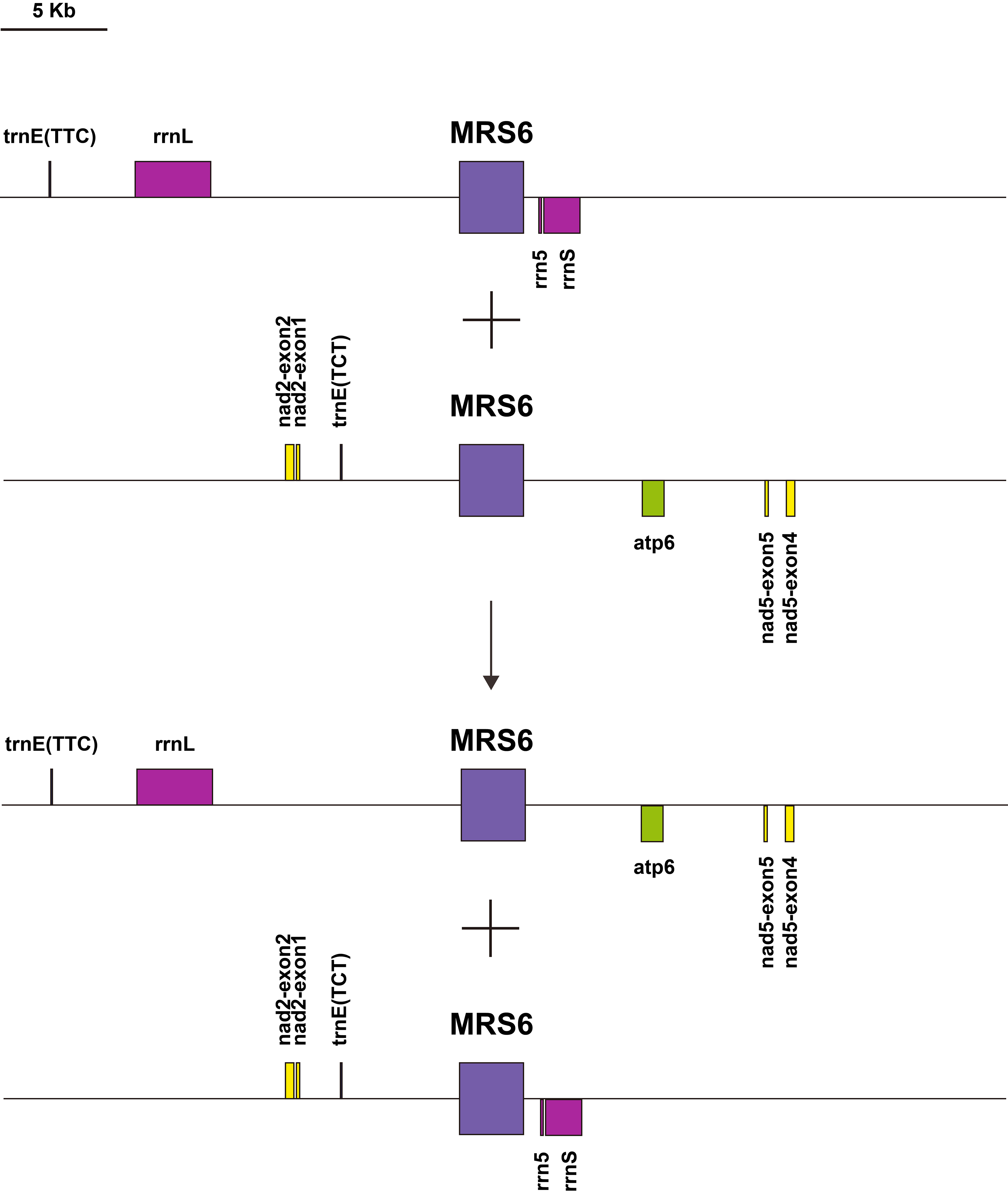

Supplement: Supplementary file 8 — Additional file 8: Fig. S8. The change of gene order based on homologous recombination. [file 12870_2020_2380_MOESM8_ESM.tif]

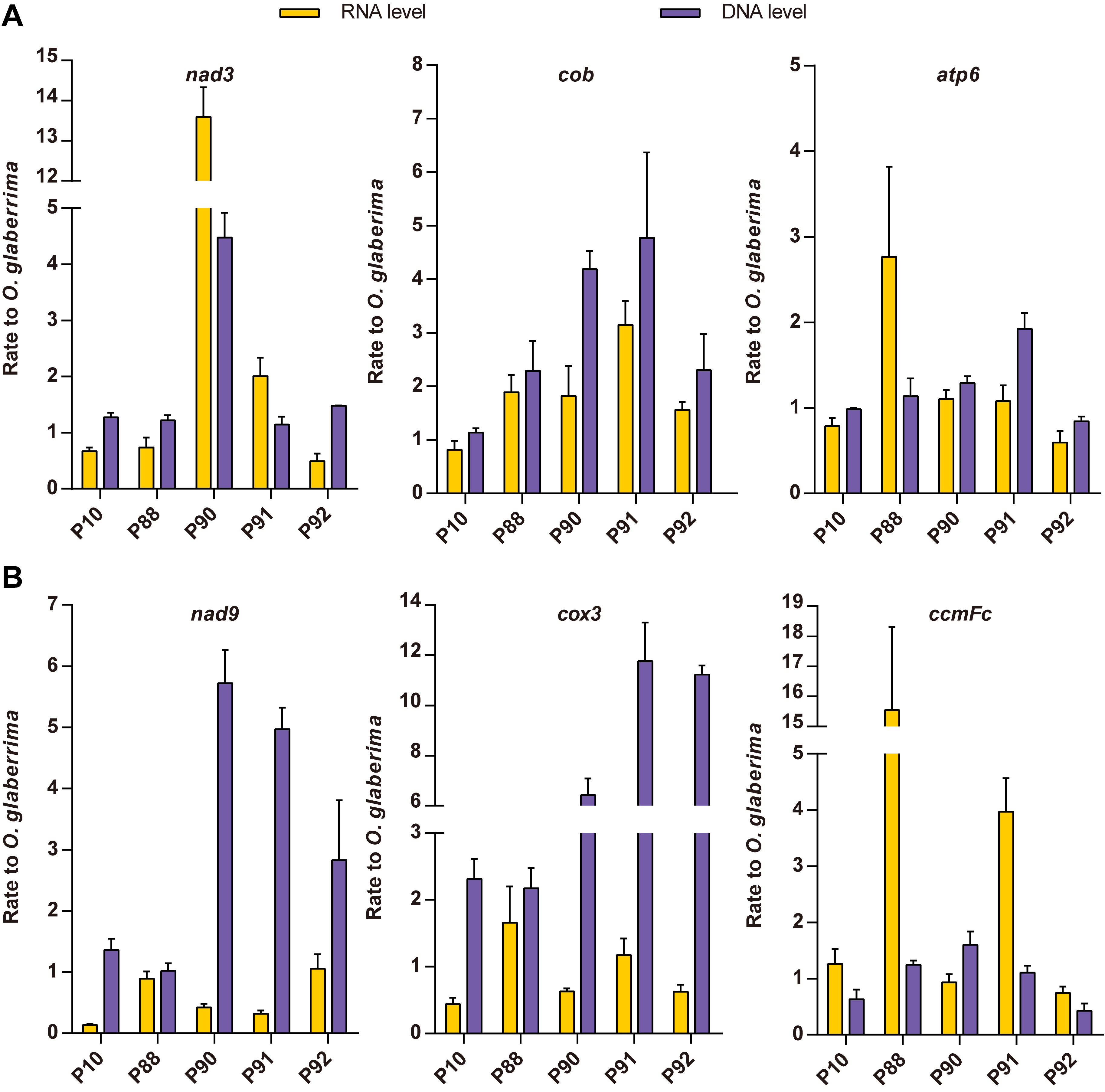

Supplement: Supplementary file 9 — Additional file 9: Fig. S9. DNA content and RNA levels of some mitochondrial genes in BIL lines compared to O. glaberrima by fluorescence quantification. A. Relative DNA and RNA level to O. glaberrima of mitochondrial genes near repeat within 2 Kb. B. Relative DNA and RNA level to O. glaberrima of mitochondrial genes far away from repeat over 2 Kb. [file 12870_2020_2380_MOESM9_ESM.tif]
