# Supplementary material for: Wide crossing diversify mitogenomes of rice
Source: BMC Plant Biol. 2020 Apr 15;20:159. doi: 10.1186/s12870-020-02380-w (PMC7160995; doi:10.1186/s12870-020-02380-w)
Supplement: Supplementary file 10 — Additional file 10: Table S1. Summary of sequencing nuclear genomes by Illumina. Table S2. Summary of sequencing mitogenomes by Illumina. Table S3. Summary of sequencing mitogenomes by PacBio. Table S4. Multi-copy genes in mitogenomes of the BIL lines. Table S5. Rearrangement types and recombination manners in BIL mitogenomes. Table S6. Variation of some representative gene clusters in mitogenome of BIL lines. Table S7. Special mitogenome sequences in 93–11. Table S8. Homologous region boundaries of BIL lines relative to O. glaberrima. Table S9. All repeats detected in O. glaberrima mitogenome. Table S10. Category of multi-copy repeats in O. glaberrima mitogenome. Table S11. RNA editing events detected in mitochondria of BIL lines. Table S12. Confirmation of RNA editing using Sanger sequencing [file 12870_2020_2380_MOESM10_ESM.docx]

**Supplementary Tables**

**Table S1** **Summary of sequencing nuclear genomes by Illumina**

| Lines | Clean reads | Clean bases (bp) | Read length (bp) | Sequencing depth (×) |
| --- | --- | --- | --- | --- |
| *O. glaberrima* | 54,089,488 | 8,113,423,200 | 150 | ~21 |
| 93-11 | 75,328,670 | 6,701,746,816 | 90 | ~17 |
| P10 | 41,128,996 | 6,169,349,400 | 150 | ~16 |
| P88 | 62,130,434 | 9,319,565,100 | 150 | ~24 |
| P90 | 53,171,266 | 7,975,689,900 | 150 | ~21 |
| P91 | 70,714,770 | 10,607,215,500 | 150 | ~28 |
| P92 | 70,274,138 | 10,541,120,700 | 150 | ~28 |

**Table S2 Summary of sequencing mitogenomes by Illumina**

| Sample | Clean reads | Clean bases (bp) | Read length (bp) | Sequencing depth (×) |
| --- | --- | --- | --- | --- |
| *O. glaberrima* | 50,237,022 | 6,279,627,750 | 125 | ~15614 |
| P10 | 97,743,331 | 12,248,672,704 | 150 | ~29170 |
| P88 | 96,957,714 | 14,543,657,100 | 150 | ~35070 |
| P90 | 143,570,300 | 21,535,545,000 | 150 | ~52583 |
| P91 | 119,223,690 | 17,883,553,500 | 150 | ~40534 |
| P92 | 110,314,382 | 16,547,157,300 | 150 | ~39126 |

**Table S3 Summary of sequencing mitogenomes by PicBio**

| Sample | Clean reads | Clean bases (bp) | Average read length (bp) | Sequencing depth (×) |
| --- | --- | --- | --- | --- |
| *O. glaberrima* | 70,871 | 853,784,994 | 12,047 | ~2123 |
| P10 | 13,512 | 1,224,879,467 | 13,512 | ~2917 |
| P88 | 62,108 | 850,286,730 | 13,690 | ~2050 |
| P90 | 115,143 | 1,543,708,510 | 13,406 | ~3769 |
| P91 | 100,414 | 1,080,046,462 | 10,755 | ~2448 |
| P92 | 45,590 | 636,685,523 | 13,965 | ~1505 |

**Table S4 Multi-copy genes in mitogenomes of the BIL lines**

| Gene | *O. glaberrima* | P10 | P88 | P90 | P91 | P92 |
| --- | --- | --- | --- | --- | --- | --- |
| *atp6* | 2 | 2 | 2 | 3 | 2 | 3 |
| *atp8* | 1 | 1 | 1 | 1 | 1 | 2 |
| *ccmC* | 1 | 1 | 1 | 1 | 2 | 2 |
| *cox2* | 1 | 2 | 2 | 1 | 1 | 1 |
| *nad6* | 1 | 1 | 1 | 1 | 2 | 2 |
| *trnR(TCT)* | 1 | 2 | 2 | 1 | 2 | 1 |
| *trnN(GTT)* | 1 | 1 | 1 | 1 | 2 | 1 |
| *trnD(GTC)* | 1 | 1 | 1 | 1 | 2 | 1 |
| *Ile-cp* | 1 | 1 | 1 | 1 | 2 | 2 |
| *trnL(AAC)* | 1 | 1 | 1 | 1 | 2 | 2 |
| *trnK(TTT)* | 1 | 1 | 1 | 1 | 2 | 1 |
| *trnM(CAT)* | 4 | 4 | 4 | 4 | 5 | 5 |
| *trnP(TGG)* | 3 | 3 | 2 | 3 | 4 | 2 |
| *rrn5* | 3 | 3 | 3 | 3 | 4 | 3 |
| *rrnS* | 3 | 3 | 3 | 3 | 4 | 3 |

**Table S5 Rearrangement types and recombination manners in BIL mitogenomes**

| Rearrangement types | | Reversal | Translocation | Fusion | Fission |
| --- | --- | --- | --- | --- | --- |
| Rearrangement event number | | 9 | 9 | 1 | 2 |
| Recombination manners | Homologous recombination | 13 | 9 | 2 | 1 |
|  | Non-homologous recombination | 5 | 9 | 0 | 3 |

**Table S6 Variation of some representative gene clusters in mitogenome of BIL lines**

| Name of gene cluster | Gene cluster in *O. glaberrima* | Gene cluster in BIL line | Detected BIL line |
| --- | --- | --- | --- |
| GJ01 | *trnE(TTC)-rrnL*-*rrn5-rrnS* | *trnE(TTC)-rrnL*-*atp6-nad5* | P10, P92 |
| GJ02 | *rrnS-rrn5*-*rrnL- trn(TTC)* | *rrnS-rrn5*-*trnP(TGG)-nad1* | P10, P88 |
| GJ03 | *nad5-atp6*-*trnR(TCT)*-*nad2* | *nad5-atp6*-*rrnL*-*trnE(TTC)* | P10, P92 |
| GJ04 | *trnC(GCA)-atp6*-*rps3*-*rpl16* | *trnC(GCA)-atp6*-*trnR(TCT)*-B | P10 |
| GJ05 | *rpl16*-*rps3*-*atp6-trnC(GCA)* | *rpl16*-*rps3-nad7*-*mttB* | P10 |
| GJ06 | *mttB*-*nad7-trnP(TGG)*-*nad1* | *mttB*-*nad7-rps3*-*rpl16* | P10 |
| GJ07 | *trnE(TTC)-rrnL*-*rrn5-rrnS* | *trnE(TTC)-rrnL*-*nad7*-*mttB* | P88 |
| GJ08 | *mttB*-*nad7-trnP(TGG)*-*nad1* | *mttB*-*nad7*-*rrnL-trnE(TTC)* | P88 |
| GJ09 | *nad5-atp6*-*trnR(TCT)*-*nad2* | *nad5-atp6*-*rps3*-*rpl16* | P90, P91 |
| GJ10 | *rps3-atp6*-*trnC(GCA)*-*ccmB* | *rps3-atp6*-*nad5-nad1* | P90, P91 |
| GJ11 | *mttB*-*nad7-trnP(TGG)*-*nad1* | *mttB*-*nad7*-*cox2-nad4* | P90 |
| GJ12 | *rps19*-*rpl2-rrnS*-*rrn5* | *rps19*-*rpl2*-*trnM(CAT)-trnD(GTC)* | P91 |
| GJ13 | *rrn5*-*rrnS*-*atp9-trnS(TGA)* | *rrn5*-*rrnS-trnP(TGG)*-B | P91 |
| GJ14 | *rrnS-rrn5*-*rrnL-trnE(TTC)* | *rrnS-rrn5-trnR(TCT)*-B | P91 |
| GJ15 | *nad2*-*trnR(TCT)*-*atp6-nad5* | *nad2*-*trnR(TCT)*-*rrn5*-*rrnS* | P91 |
| GJ16 | *trnC(GCA)-atp6*-*rps3*-*rpl16* | *trnC(GCA)-atp6*-*cox2-nad4* | P91 |
| GJ17 | *trnD(GTC)-trnM(CAT)*- *ccmC*-*Ile-cp* | *trnD(GTC)-trnM(CAT)*-*rpl2*-*rps19* | P91 |
| GJ18 | *cox3*-*trnP(TGG)*-*rrn5-rrnS* | *cox3*-*trnP(TGG)-nad7*-*mttB* | P91, P92 |
| GJ19 | *rrnS-rrn5*-*rrnL-trnE(TTC)* | *rrnS-rrn5*-*cox2-nad4* | P92 |
| GJ20 | *rpl16*-*rps3*-*atp6-trnC(GCA)* | *rpl16*-*rps3*-*rrn5-rrnS* | P92 |

Note: B represents the boundary of scaffold.

**Table S7 Special mitogenome sequences in 93-11**

| Sequence No. | Start site | End site | Size (bp) |
| --- | --- | --- | --- |
| SS9-1 | 1 | 731 | 731 |
| SS9-2 | 775 | 6551 | 5777 |
| SS9-3 | 19054 | 19553 | 500 |
| SS9-4 | 92311 | 95792 | 3482 |
| SS9-5 | 95836 | 101611 | 5776 |
| SS9-6 | 114114 | 114613 | 500 |
| SS9-7 | 142237 | 149275 | 7039 |
| SS9-8 | 194613 | 195429 | 817 |
| SS9-9 | 468562 | 472295 | 3734 |

**Table S8 Homologous region boundaries of BIL lines relative to *O*. *glaberrima***

| BIL lines | Position in BIL lines | | Position in *O. glaberrima* | |
| --- | --- | --- | --- | --- |
| P10 | 3549 | 66010 | -229663 | -292123 |
|  | 67273 | 153580 | 309327 | 395634 |
|  | 157407 | 216513 | 123241 | 182346 |
|  | 217687 | 334794 | -1500 | -118604 |
|  | 367354 | 413300 | 182682 | 228628 |
| P88 | 5452 | 124757 | 51 | 119353 |
|  | 124851 | 211115 | 309327 | 395583 |
|  | 213005 | 384538 | 121253 | 292784 |
| P90 | 15172 | 67579 | 126567 | 178973 |
|  | 75886 | 134878 | 233003 | 291996 |
|  | 135011 | 221276 | 309327 | 395585 |
|  | 221346 | 221376 | 395654 | 395684 |
|  | 227813 | 339151 | 8132 | 119467 |
|  | 348511 | 400271 | -178978 | -230738 |
| P91 | 7650 | 66833 | 121312 | 180494 |
|  | 71330 | 130551 | 230714 | 289935 |
|  | 132644 | 179123 | -182021 | -228500 |
|  | 206464 | 252805 | 341627 | 387968 |
|  | 260504 | 279676 | 309327 | 328499 |
|  | 293254 | 395898 | 14559 | 117204 |
|  | 420592 | 433649 | -1500 | -14554 |
| P92 | 4820 | 123427 | 3 | 118606 |
|  | 123428 | 188339 | -118607 | -183517 |
|  | 189842 | 251884 | -227744 | -289785 |
|  | 273918 | 302414 | 185020 | 213516 |
|  | 336404 | 373549 | 350878 | 388023 |
|  | 381193 | 413587 | 309327 | 341721 |

Note: All scaffolds were artificially connected.

**Table S9 All repeats detected in *O. glaberrima* mitogenome**

| Repeat name | Scaffold | Start site | End site | Length | Distance from the nearest genes |
| --- | --- | --- | --- | --- | --- |
| LR01 | 1 | 219746 | 230741 | 10996 | >2 Kb |
| LR02 | 1 | 289789 | 300767 | 10979 | >2 Kb |
| LR03 | 1 | 178976 | 186107 | 7132 | ≤2 Kb |
| LR04 | 1 | 288699 | 295828 | 7130 | >2 Kb |
| LR05 | 1 | 112988 | 120103 | 7116 | >2 Kb |
| LR06 | 1 | 227745 | 234860 | 7116 | >2 Kb |
| LR07 | 2 | 94721 | 101408 | 6688 | >2 Kb |
| LR08 | 1 | 1498 | 8183 | 6686 | >2 Kb |
| LR09 | 1 | 119267 | 123238 | 3972 | >2 Kb |
| LR10 | 2 | 94721 | 98692 | 3972 | >2 Kb |
| LR11 | 1 | 117107 | 120103 | 2997 | >2 Kb |
| LR12 | 1 | 182021 | 185017 | 2997 | ≤2 Kb |
| LR13 | 1 | 182021 | 184355 | 2335 | ≤2 Kb |
| LR14 | 1 | 227745 | 230079 | 2335 | >2 Kb |
| LR15 | 1 | 1 | 2334 | 2334 | >2 Kb |
| R1 | 1 | 182021 | 182857 | 837 | ≤2 Kb |
| R2 | 1 | 227745 | 228581 | 837 | >2 Kb |
| R3 | 1 | 291949 | 292785 | 837 | >2 Kb |
| R4 | 2 | 94721 | 95557 | 837 | >2 Kb |
| IR01 | 1 | 14107 | 14556 | 450 | ≤2 Kb |
| IR02 | 2 | 40859 | 41308 | 450 | ≤ 2 Kb |
| IR03 | 1 | 1498 | 1675 | 178 | >2 Kb |
| IR04 | 1 | 119267 | 119444 | 178 | >2 Kb |
| IR05 | 1 | 182680 | 182857 | 178 | >2 Kb |
| IR06 | 1 | 228404 | 228581 | 178 | >2 Kb |
| IR07 | 1 | 291949 | 292126 | 178 | >2 Kb |
| IR08 | 2 | 8381 | 8558 | 178 | ≤2 Kb |
| IR09 | 1 | 1448 | 1590 | 143 | >2 Kb |
| IR10 | 1 | 119217 | 119359 | 143 | >2 Kb |
| IR11 | 1 | 136164 | 136306 | 143 | >2 Kb |
| IR12 | 1 | 291899 | 292041 | 143 | >2 Kb |
| IR13 | 1 | 155573 | 155692 | 120 | ≤2 Kb |
| IR14 | 1 | 255859 | 255978 | 120 | ≤2 Kb |
| IR15 | 1 | 72679 | 72797 | 119 | ≤2 Kb |
| IR16 | 1 | 255100 | 255218 | 119 | ≤2 Kb |
| IR17 | 1 | 189699 | 189815 | 117 | ≤2 Kb |
| IR18 | 1 | 245526 | 245642 | 117 | ≤2 Kb |
| IR19 | 2 | 12353 | 12469 | 117 | ≤2 Kb |
| IR20 | 2 | 85328 | 85443 | 116 | ≤2 Kb |
| IR21 | 1 | 165291 | 165399 | 109 | ≤2 Kb |
| IR22 | 1 | 195124 | 195232 | 109 | >2 Kb |
| IR23 | 1 | 295865 | 295973 | 109 | >2 Kb |
| IR24 | 1 | 16411 | 16517 | 107 | ≤2 Kb |
| IR25 | 1 | 103539 | 103637 | 99 | >2 Kb |
| IR26 | 2 | 7024 | 7118 | 95 | ≤2 Kb |
| IR27 | 2 | 93364 | 93458 | 95 | >2 Kb |
| IR28 | 1 | 136164 | 136256 | 93 | >2 Kb |
| IR29 | 2 | 8381 | 8473 | 93 | ≤2 Kb |
| IR30 | 2 | 94721 | 94813 | 93 | >2 Kb |
| IR31 | 1 | 57312 | 57393 | 82 | ≤2 Kb |
| IR32 | 2 | 41227 | 41308 | 82 | ≤2 Kb |
| IR33 | 1 | 62079 | 62156 | 78 | ≤2 Kb |
| IR34 | 1 | 74840 | 74917 | 78 | ≤2 Kb |
| IR35 | 1 | 99788 | 99859 | 72 | >2 Kb |
| IR36 | 1 | 259194 | 259265 | 72 | >2 Kb |
| IR37 | 1 | 256799 | 256869 | 71 | ≤2 Kb |
| IR38 | 2 | 61175 | 61245 | 71 | >2 Kb |
| IR39 | 1 | 136300 | 136366 | 67 | >2 Kb |
| IR40 | 1 | 204051 | 204117 | 67 | ≤2 Kb |
| IR41 | 1 | 238385 | 238451 | 67 | >2 Kb |
| IR42 | 1 | 242845 | 242911 | 67 | ≤2 Kb |
| IR43 | 1 | 23932 | 23997 | 66 | ≤2 Kb |
| IR44 | 1 | 71030 | 71095 | 66 | ≤2 Kb |
| IR45 | 1 | 142677 | 142742 | 66 | ≤2 Kb |
| IR46 | 1 | 204052 | 204117 | 66 | ≤2 Kb |
| IR47 | 1 | 204053 | 204118 | 66 | ≤2 Kb |
| IR48 | 1 | 210932 | 210997 | 66 | >2 Kb |
| IR49 | 1 | 238385 | 238450 | 66 | >2 Kb |
| IR50 | 1 | 242845 | 242910 | 66 | ≤2 Kb |
| IR51 | 2 | 37757 | 37822 | 66 | >2 Kb |
| IR52 | 2 | 58001 | 58066 | 66 | ≤2 Kb |
| IR53 | 1 | 4965 | 5029 | 65 | >2 Kb |
| IR54 | 1 | 96282 | 96346 | 65 | >2 Kb |
| IR55 | 1 | 122734 | 122798 | 65 | >2 Kb |
| IR56 | 1 | 142677 | 142741 | 65 | ≤2 Kb |
| IR57 | 1 | 238385 | 238449 | 65 | >2 Kb |
| IR58 | 2 | 37755 | 37819 | 65 | >2 Kb |
| IR59 | 2 | 37758 | 37822 | 65 | >2 Kb |
| IR60 | 2 | 58002 | 58066 | 65 | ≤2 Kb |
| IR61 | 2 | 58003 | 58067 | 65 | ≤2 Kb |
| IR62 | 1 | 4966 | 5029 | 64 | >2 Kb |
| IR63 | 1 | 27089 | 27152 | 64 | >2 Kb |
| IR64 | 1 | 71032 | 71095 | 64 | ≤2 Kb |
| IR65 | 1 | 93419 | 93482 | 64 | >2 Kb |
| IR66 | 1 | 96283 | 96346 | 64 | >2 Kb |
| IR67 | 1 | 122735 | 122798 | 64 | >2 Kb |
| IR68 | 1 | 136303 | 136366 | 64 | >2 Kb |
| IR69 | 1 | 142678 | 142741 | 64 | ≤2 Kb |
| IR70 | 1 | 204054 | 204117 | 64 | ≤2 Kb |
| IR71 | 1 | 242845 | 242908 | 64 | ≤2 Kb |
| IR72 | 2 | 37756 | 37819 | 64 | >2 Kb |
| IR73 | 2 | 37758 | 37821 | 64 | >2 Kb |
| IR74 | 2 | 98189 | 98252 | 64 | >2 Kb |
| IR75 | 1 | 4967 | 5029 | 63 | >2 Kb |
| IR76 | 1 | 35006 | 35068 | 63 | ≤2 Kb |
| IR77 | 1 | 99427 | 99489 | 63 | >2 Kb |
| IR78 | 1 | 122736 | 122798 | 63 | >2 Kb |
| IR79 | 1 | 142680 | 142742 | 63 | ≤2 Kb |
| IR80 | 1 | 204056 | 204118 | 63 | ≤2 Kb |
| IR81 | 1 | 242846 | 242908 | 63 | ≤2 Kb |
| IR82 | 2 | 41794 | 41856 | 63 | ≤2 Kb |
| IR83 | 2 | 86019 | 86081 | 63 | ≤2 Kb |
| IR84 | 2 | 98190 | 98252 | 63 | >2 Kb |
| IR85 | 1 | 4968 | 5029 | 62 | >2 Kb |
| IR86 | 1 | 71032 | 71093 | 62 | ≤2 Kb |
| IR87 | 1 | 96283 | 96344 | 62 | >2 Kb |
| IR88 | 1 | 122737 | 122798 | 62 | >2 Kb |
| IR89 | 1 | 238385 | 238446 | 62 | >2 Kb |
| IR90 | 2 | 58005 | 58066 | 62 | ≤2 Kb |
| IR91 | 2 | 98191 | 98252 | 62 | >2 Kb |
| IR92 | 1 | 97179 | 97237 | 59 | >2 Kb |
| IR93 | 2 | 18352 | 18410 | 59 | ≤2 Kb |
| IR94 | 1 | 18483 | 18536 | 54 | ≤2 Kb |
| IR95 | 2 | 43609 | 43662 | 54 | ≤2 Kb |
| IR96 | 1 | 281661 | 281713 | 53 | ≤2 Kb |
| IR97 | 1 | 296037 | 296089 | 53 | >2 Kb |
| IR98 | 1 | 95745 | 95794 | 50 | >2 Kb |
| IR99 | 1 | 233065 | 233114 | 50 | >2 Kb |

Note: Distance means the repeat to the up- or downstream nearest gene body.

**Table S10 Category of multi-copy repeats in *O. glaberrima* mitogenome**

| Repeat | Length (bp) | Located scaffold | Start site | End site |
| --- | --- | --- | --- | --- |
| MRS1-1 | 10996 | 1 | 219746 | 230741 |
| MRS1-2 | 10979 | 1 | 289789 | 300767 |
| MRS2-1 | 7132 | 1 | 178976 | 186107 |
| MRS2-2 | 7130 | 1 | 288699 | 295828 |
| MRS3-1 | 7116 | 1 | 112988 | 120103 |
| MRS3-2 | 7116 | 1 | 227745 | 234860 |
| MRS4-1 | 6686 | 1 | 1498 | 8183 |
| MRS4-2 | 6688 | 2 | 94721 | 101408 |
| MRS5-1 | 3972 | 1 | 119267 | 123238 |
| MRS5-2 | 3972 | 2 | 94721 | 98692 |
| MRS6-1 | 2997 | 1 | 117107 | 120103 |
| MRS6-2 | 2997 | 1 | 182021 | 185017 |
| MRS7-1 | 2334 | 1 | 1 | 2334 |
| MRS7-2 | 2335 | 1 | 182021 | 184355 |
| MRS7-3 | 2335 | 1 | 227745 | 230079 |
| MRS8-1 | 837 | 1 | 182021 | 182857 |
| MRS8-2 | 837 | 2 | 94721 | 95557 |
| MRS9-1 | 450 | 1 | 14107 | 14556 |
| MRS9-2 | 450 | 2 | 40859 | 41308 |
| MRS10-1 | 178 | 1 | 1498 | 1675 |
| MRS10-2 | 178 | 2 | 8381 | 8558 |
| MRS11-1 | 143 | 1 | 1448 | 1590 |
| MRS11-2 | 143 | 1 | 136164 | 136306 |
| MRS12-1 | 120 | 1 | 155573 | 155692 |
| MRS12-2 | 120 | 1 | 255858 | 255977 |
| MRS13-1 | 119 | 1 | 72679 | 72797 |
| MRS13-2 | 119 | 1 | 255099 | 255217 |
| MRS14-1 | 117 | 1 | 189699 | 189815 |
| MRS14-2 | 117 | 2 | 12353 | 12469 |
| MRS15-1 | 117 | 1 | 245525 | 245641 |
| MRS15-2 | 116 | 2 | 85328 | 85443 |
| MRS16-1 | 107 | 1 | 16411 | 16517 |
| MRS16-2 | 109 | 1 | 195124 | 195232 |
| MRS17-1 | 109 | 1 | 165291 | 165399 |
| MRS17-2 | 109 | 1 | 295865 | 295973 |
| MRS18-1 | 99 | 1 | 103539 | 103637 |
| MRS18-2 | 95 | 2 | 7024 | 7118 |
| MRS19-1 | 93 | 1 | 136164 | 136256 |
| MRS19-2 | 93 | 2 | 8381 | 8473 |
| MRS19-3 | 93 | 2 | 94721 | 94813 |
| MRS20-1 | 82 | 1 | 57312 | 57393 |
| MRS20-2 | 82 | 2 | 41227 | 41308 |
| MRS21-1 | 78 | 1 | 62079 | 62156 |
| MRS21-2 | 78 | 1 | 74840 | 74917 |
| MRS22-1 | 72 | 1 | 99788 | 99859 |
| MRS22-2 | 72 | 1 | 259193 | 259264 |
| MRS23-1 | 71 | 1 | 256798 | 256868 |
| MRS23-2 | 71 | 2 | 61175 | 61245 |
| MRS24-1 | 67 | 1 | 136300 | 136366 |
| MRS24-2 | 67 | 1 | 242844 | 242910 |
| MRS25-1 | 67 | 1 | 204050 | 204116 |
| MRS25-2 | 67 | 1 | 238384 | 238450 |
| MRS26-1 | 66 | 1 | 23932 | 23997 |
| MRS26-2 | 66 | 1 | 210931 | 210996 |
| MRS27-1 | 64 | 1 | 27089 | 27152 |
| MRS27-2 | 64 | 1 | 93419 | 93482 |
| MRS28-1 | 63 | 1 | 35006 | 35068 |
| MRS28-2 | 63 | 2 | 41794 | 41856 |
| MRS29-1 | 63 | 1 | 99427 | 99489 |
| MRS29-2 | 63 | 2 | 86019 | 86081 |
| MRS30-1 | 62 | 1 | 4968 | 5029 |
| MRS30-2 | 62 | 1 | 71032 | 71093 |
| MRS30-3 | 62 | 1 | 96283 | 96344 |
| MRS30-4 | 62 | 1 | 136303 | 136364 |
| MRS30-5 | 62 | 1 | 142680 | 142741 |
| MRS30-6 | 62 | 1 | 204055 | 204116 |
| MRS30-7 | 62 | 1 | 238384 | 238445 |
| MRS30-8 | 62 | 1 | 242846 | 242907 |
| MRS30-9 | 62 | 2 | 37758 | 37819 |
| MRS30-10 | 62 | 2 | 58005 | 58066 |
| MRS31-1 | 54 | 1 | 18483 | 18536 |
| MRS31-2 | 54 | 2 | 43609 | 43662 |
| MRS32-1 | 53 | 1 | 281660 | 281712 |
| MRS32-2 | 53 | 1 | 296037 | 296089 |
| MRS33-1 | 50 | 1 | 95745 | 95794 |
| MRS33-2 | 50 | 1 | 233065 | 233114 |

**Table S11 RNA editing events detected in mitochondria of BIL lines**

| Category | Editing events | Number | Percentage |
| --- | --- | --- | --- |
| Nucleotide transition | C-to-U | 525 | 99.2% |
|  | U-to-C | 4 | 0.8% |
| Edited triplet site | 1st | 180 | 34.0% |
|  | 2nd | 281 | 53.1% |
|  | 3rd | 68 | 12.9% |
| Amino acid alteration | Synonymous | 70 | 13.2% |
|  | Nonsynonymous | 459 | 86.8% |

**Table S12 Confirmation of RNA editing using Sanger sequencing**

| Examined editing sites | *O. glaberrima* | | | BIL lines | | | Tested line |
| --- | --- | --- | --- | --- | --- | --- | --- |
|  | Editing rate by RNA-Seq | Edited clone | Editing rate | Editing rate by RNA-Seq | Edited clone | Editing rate |  |
| cox2-144 | 90.4% | 46/50 | 92% | 28.7% | 23/50 | 46% | P91 |
| mttB-354 | 4.4% | 2/50 | 4% | 54.2% | 21/50 | 42% | P92 |
| nad7-1110 | 7.9% | 4/50 | 8% | 91.0% | 35/50 | 70% | P10 |

Note: In the Sanger sequencing, each editing site were sequenced 50 separate clones.
