# Supplementary material for: Wide crossing diversify mitogenomes of rice
Source: BMC Plant Biol. 2020 Apr 15;20:159. doi: 10.1186/s12870-020-02380-w (PMC7160995; doi:10.1186/s12870-020-02380-w)
Supplement: Supplementary file 11 — Additional file 11 Supplementary information 1 [file 12870_2020_2380_MOESM11_ESM.docx]

**All primers used in the experiment**

| Name | Sequence (5’ to 3’) |
| --- | --- |
| ds1-F | CAAGCACAACCTCCTAAA |
| ds1-R | TGTTCCGATCCTATTTCC |
| ds2-F | TGCGACTCGGAAGGTATG |
| ds2-R | AATGTCCCGTCCACCAAT |
| ds3-F | AAATGAGCGTTAGATGTTG |
| ds3-R | ATGGCAGTTGGATAGTAGAT |
| ds4-F | AAGTATCTATCGCTATGTGC |
| ds4-R | CAGACGAAAGGAATGAAA |
| ds5-F | ACCCGAGGCAACTAAACT |
| ds5-R | GCAAATTGGATGCGTAAA |
| ds6-F | CGAGCCAATGCCAATACC |
| ds6-R | ACCTCTTCGGGACCTGTT |
| ds7-F | TTCGCTCGTCATTTATTC |
| ds7-R | CCTTACCGCTTTACACCT |
| ds8-F | TATGAAAGCAGGAATCGC |
| ds8-R | AGAGCACCCAGTCACAGC |
| ds9-F | GGAATCACGGTATCAGTA |
| ds9-R | CCTTATGGGCAAGAACAC |
| ds10-F | CCTTTCACATGGGCATTA |
| ds10-R | AGAACCGCACTCAACTCA |
| ds11-F | CTTACCTTTGGTGGCATTG |
| ds11-R | AGTGGAATCACCCGTTGG |
| ds12-F | GCTAAGGCATACTTCACTG |
| ds12-R | TTCTGCTGCTTGTCTCCT |
| ds13-F | AGTTGAAGCGTTCCTACC |
| ds13-R | AGTCGAGCTGGATGAGTT |
| ds14-F | GAAAGGAAAGGCAGGACA |
| ds14-R | CAATGGAAGATCGGGACT |
| ds15-F | GGCGAGCATAAGAACCAT |
| ds15-R | GGACAACTCAAGCACCAA |
| ds16-F | ACAGAACAAGAGGGAAAGACC |
| ds16-R | CGTGGCGTGAGTTGGAAT |
| ds17-F | AAAAGTTACCCATATCAAGACC |
| ds17-R | TAATCCACAAACCATCCC |
| ds18-F | GAGTGGAATCACCCGTTGG |
| ds18-R | ATGGGATGATCCTTACCTTTGG |
| ds19-F | TATTGTCCGCTCACTTCC |
| ds19-R | CTTCTTGTTTAGGGTCTTG |
| ds20-F | CGAGACCTTGTGCCTTCC |
| ds20-R | ACCTTGCGTCATCCTTCG |
| ds21-F | ACAGATGCGCTGACCAGA |
| ds21-R | AAGGCGTACATGAGTTCCTC |
| MRS8ab-F | TCGAGCGACCTGGATTAT |
| MRS8ab-R | CCCTTTCGAGAAATGGTG |
| MRS8cd-F | GAAATTGTTCGGGAGACA |
| MRS8cd-R | CGCTTTAGGCACAGGGAG |
| MRS9ab-F | CGGAAGAATAGGTGAGTG |
| MRS9ab-R | ATGCCAATAGACGAAGTG |
| MRS9cd-F | CTGTTTCCCTAGTTTACC |
| MRS9cd-R | AATCCTCTATTTCCTGCT |
| MRS10ab-F | CCGAATTAGCGGCTGTAC |
| MRS10ab-R | GACTGCCATCGCCATCTC |
| MRS10cd-F | ACAGATGCGCTGACCAGA |
| MRS10cd-R | GGTCTACAGTAGTGCCTTGC |
| MRS11ab-F | CTTTCGAGAAATGGTGGC |
| MRS11ab-R | TTCTGGCTGGTTAGAGCA |
| MRS11cd-F | TGGTCCTCGGAACCCTAC |
| MRS11cd-R | GATGTTTGAACTTATTAGCG |
| MRS12ab-F | CGGCGAGTGGAAGAGTTG |
| MRS12ab-R | TGCGGCGTTGGATTAGAG |
| MRS12cd-F | TCAAATCAAACTTTTCTTGGAA |
| MRS12cd-R | TCCCGAGATACTGGTGGT |
| MRS13ab-F | AAGCGATCACGAACGAAG |
| MRS13ab-R | TGGCTGCTACAAGAATGG |
| MRS13cd-F | CATTCTTAGTTGATTCCACGAT |
| MRS13cd-R | AAGGAAAGGCAGGACAGC |
| MRS14ab-F | AAGGAGCGGCAAATAAGT |
| MRS14ab-R | ATGTGGTTCCGAGTTTCG |
| MRS14cd-F | GCATCAATCATCGCTCAG |
| MRS14cd-R | ACGTTCCACCACTTCTCC |
| MRS15ab-F | TCTGTTCGTCGGTCCTTG |
| MRS15ab-R | TTTCATAGCCGTTTCTCG |
| MRS15cd-F | AACGCTTTCTAAATAGTGAC |
| MRS15cd-R | ACCTAAACTCTTCCGACT |
| MRS16ab-F | CCCCGTTTGCCTTTACTA |
| MRS16ab-R | GACGCCTTTCCCTTTGTA |
| MRS16cd-F | ATGGCACCCAATACTGAA |
| MRS16cd-R | ATTAGCCAGCCTCATCCA |
| MRS17ab-F | CGGTATACTGTACTAATATGTG |
| MRS17ab-R | ACATTATAGCCAGCAACT |
| MRS17cd-F | AAAGTATCATGTTGCTCCTC |
| MRS17cd-R | AACGAAGATTTGATTGGTTT |
| MRS18ab-F | AACAGAACTTCGATAGGC |
| MRS18ab-R | CAACTTTACAAATTCGCT |
| MRS18cd-F | ACAGGAGCACCATCTTAA |
| MRS18cd-R | TTCGATTTTCTATTCACTCA |
| MRS20ab-F | AGGTAAAGCGAAGCACAG |
| MRS20ab-R | AGCCGAACGACTACTAAATG |
| MRS20cd-F | ACTCTTCATCAATCCCTAC |
| MRS20cd-R | TCCTCTATTTCCTGCTCT |
| MRS21ab-F | GCGTTGTAGTATGGGTGA |
| MRS21ab-R | TGAGGAAGGAAGGGGTAG |
| MRS21cd-F | AACGTCCACTTTGCATTT |
| MRS21cd-R | CTCTTTCGTGATTTTGTAG |
| MRS22ab-F | ACAGTCCCATAAGCAGTAG |
| MRS22ab-R | TTCCCTTGGATCAGATAG |
| MRS22cd-F | AATAACTTACGGCGAGAC |
| MRS22cd-R | GGATAGAACCAGCGTTTA |
| MRS23ab-F | AAAGAGGCAAAGTGATGC |
| MRS23ab-R | CGAATTTACCTGCGAATAA |
| MRS23cd-F | ATCTGATAAGGTTGGGTTAA |
| MRS23cd-R | GTTTCTAGCAAGCGAGTT |
| MRS24ab-F | CCTCAGAACGACCTTCAC |
| MRS24ab-R | TGGAATGCCTAGTTTGACT |
| MRS24cd-F | CGTGCTAGATAGTCACCCAT |
| MRS24cd-R | ACCACCTGAGCGAACCAA |
| MRS25ab-F | GAACTGTCACGCAGAAGT |
| MRS25ab-R | TGGGTATTGTATCCCTTT |
| MRS25cd-F | ATTCCATACCTTCCACCGACTT |
| MRS25cd-R | TGCGGATTTGAACCAGTGTC |
| MRS26ab-F | CTCTTCCAACTCGTCCCG |
| MRS26ab-R | TGCTAATCCGATGCCCAC |
| MRS26cd-F | GATTGACAGATGGCTCCG |
| MRS26cd-R | GAATGCTCCTCCTCGTAG |
| MRS27ab-F | TGCTTCATATCTTCAAGG |
| MRS27ab-R | AATCCGAAATAACAATCA |
| MRS27cd-F | TTCCTTACGCAAGTTAGAGC |
| MRS27cd-R | ATCGCAAGCAAGTAAACC |
| MRS28ab-F | CTTCCTGGCACATACATTA |
| MRS28ab-R | TCTATGCACATCGCTCTTT |
| MRS28cd-F | AACTAACGATTATGCTGCTC |
| MRS28cd-R | CACAAATTCTTTAGGACCAG |
| MRS29ab-F | CGCAGACTCACTTGGTAA |
| MRS29ab-R | GACACCGATTCATTTCTA |
| MRS29cd-F | ATTCGGGAAGGCTTGATA |
| MRS29cd-R | CCGTTATGCCCTCGTTAG |
| MRS31ab-F | TGAAGAAGACTATCGCACCA |
| MRS31ab-R | CTTGCCGATCACATCCAA |
| MRS31cd-F | TTTATACCTACTATTCCGTCTG |
| MRS31cd-R | AATCCAAGGCATTTCGTC |
| MRS32ab-F | CATCGGATAGCTTTCTTG |
| MRS32ab-R | TACACTGGAGCGTTTGTC |
| MRS32cd-F | GCTAAGGCATACTTCACTG |
| MRS32cd-R | AAGAACATAACCAATACGG |
| MRS33ab-F | CGAGTGCCCTTTGTAAGT |
| MRS33ab-R | GTGTTCATGTTGGCGATT |
| MRS33cd-F | GACAGCGGACGAACAGAG |
| MRS33cd-R | AGCGGGAATGAGAACACC |
| action-F | AGACCACCTACAACTCTATCA |
| action-R | TTCCTCCACTAAGCACAA |
| nad3-F | GTGATGCCAGAAGTCGTT |
| nad3-R | TAGGAGGTACTGCCCAAG |
| cob-F | CGACCGATTCACCAAGGA |
| cob-R | TGGGCGTTATGGCAAAGA |
| atp6-F | ATTTGGTCTTGATATGGGTA |
| atp6-R | ATTTGGCACTGACTTTCC |
| nad9-F | CGGTAGTCAGTCCATTTC |
| nad9-R | CATAGCGTACTTCCACATA |
| cox3-F | GTAGCAACCGTTTCACTG |
| cox3-R | ATTGGCGAATACCACATA |
| ccmFc-F | TCGTTTCGTTCCCGTTCT |
| ccmFc -R | CACGCTTCGCTGACCTAT |
| cox2-144-F | AAGACGCTGCAACACCTA |
| cox2-144-R | CACCGCTTCTACGACGAT |
| mttB-354-F | ACCCTTTCTTACCCTACC |
| mttB-354-R | CCTCATTCTAATCGTCCAG |
| nad7-1110-F | ATACGACCTGGTGGAGTG |
| nad7-1110-R | CATCTGCTGGCATGTGAT |
